# miR156a‐targeted SBP‐Box transcription factor SlSPL13 regulates inflorescence morphogenesis by directly activating *SFT* in tomato

**DOI:** 10.1111/pbi.13331

**Published:** 2020-01-25

**Authors:** Long Cui, Fangyan Zheng, Jiafa Wang, Chunli Zhang, Fangming Xiao, Jie Ye, Changxing Li, Zhibiao Ye, Junhong Zhang

**Affiliations:** ^1^ Key Laboratory of Horticultural Plant Biology Ministry of Education Huazhong Agricultural University Wuhan China; ^2^ Department of Plant Sciences University of Idaho Moscow ID USA

**Keywords:** SPL13, *SFT*, inflorescence structure, plant architecture, yield, tomato

## Abstract

The inflorescences and lateral branches of higher plants are generated by lateral meristems. The structure of the inflorescence has a direct effect on fruit yield in tomato (*Solanum lycopersicum*). We previously demonstrated that miR156a plays important roles in determining the structures of the inflorescences and lateral branches in tomato by suppressing the expression of the *SQUAMOSA PROMOTER BINDING PROTEIN LIKE (SPL)* transcription factor gene family. However, information on regulatory pathways associated with inflorescence morphogenesis is still lacking. In this study, we demonstrate that *SPL13* is the major *SPL* involved in miR156a‐regulated tomato inflorescence structure determination and lateral branch production. Suppressing the expression of *SPL13* in tomato increases the number of inflorescences on vegetative branches and lateral branches, decreases the number of flowers and fruit, and reduces fruit size and yield. Genetic and biochemical evidence indicate that SPL13 controls inflorescence development by positively regulating the expression of the tomato inflorescence‐associated gene *SINGLE FLOWER TRUSS* (*SFT*) by directly binding to its promoter region. Thus, our findings provide a major advance to our understanding of the miR156a‐SlSPL‐based mechanism that regulates plant architecture and yield in tomato.

## Introduction

Plant architecture is an important determinant of crop yield. The architecture of higher plants is established by lateral meristems, which fundamentally influences the lives of plants (Gallavotti *et al.*, 2004; Martin‐Trillo *et al.*, 2011). Recent studies have shown that the SPL transcription factors associated with SPL transcription factor‐binding proteins regulate the architecture of rice, bread wheat, soybean and maize (Chuck *et al.*, 2014; Du *et al.*, 2017, 2017; Liu *et al.*, 2017; Song *et al.*, 2017; Wang and Zhang, 2017). In rice, the SPL transcription factor IPA1 regulates plant architecture by influencing rice tillering (Jiao *et al.*, 2010; Lu *et al.*, 2013; Miura *et al.*, 2010). The miR156‐TaSPL3/17 module can regulate plant architecture in bread wheat (Liu *et al.*, 2017). In soybean, GmSPL9d, the target of GmmiR156b, regulates axillary bud formation and branching by physically interacting with WUSCHEL (WUS) (Sun *et al.*, 2019). In switchgrass, the *miR156‐SPL4* module predominantly regulates axillary bud formation (Gou *et al.*, 2017). In maize, SBP‐box transcription factor genes *UNBRANCHED3 (UB2)* and *UNBRANCHED3 (UB3)* affect yield traits by regulating the rate of lateral primordia initiation (Chuck *et al.*, 2014; Du *et al.*, 2017, 2017). Thus, the *SPL* genes encode important regulators of plant architecture traits in crop plants.

In addition to affecting plant architecture, *SPL* genes affect other aspects of plant development. In Arabidopsis, AtSPL9 and AtSPL15 regulate shoot maturation, plastochron length and organ size (Gou *et al.*, 2011; Hyun *et al.*, 2016; Schwarz *et al.*, 2008; Usami *et al.*, 2009; Wang *et al.*, 2009; Wang *et al.*, 2008; Wu *et al.*, 2009; Yu *et al.*, 2010; Yu *et al.*, 2015b; Zhang *et al.*, 2015). AtSPL3 regulates shoot development and flowering (Gandikota *et al.*, 2007; Wu and Poethig, 2006). In the past few years, a number of *SPL* genes that control yield traits have been cloned from rice (Jiao *et al.*, 2010; Miura *et al.*, 2010; Si *et al.*, 2016; Wang *et al.*, 2012). *GLW7* encodes OsSPL13 in rice, which positively regulates cell size in the grain hull. Indeed, overexpression of *OsSPL13* increased rice grain length and yield (Si *et al.*, 2016). The rice *IDEAL PLANT ARCHITECTURE1 (IPA1)* gene *OsSPL14* regulates plant architecture by controlling the number of tillers and panicle branches (Jiao *et al.*, 2010; Miura *et al.*, 2010). The quantitative trait locus *GW8* (*OsSPL16*) regulates rice grain size, shape and quality (Wang *et al.*, 2015; Wang *et al.*, 2012). In switchgrass, the miR156‐targeted *SPL7* and *SPL8* regulate inflorescence development and play key roles in phase transitions and flowering (Gou *et al.*, 2019; Hardin *et al.*, 2013). In alfalfa, miR156‐targeted *MsSPL13* regulates vegetative and reproductive development (Gao *et al.*, 2016; Gao *et al.*, 2018). Another miR156‐targeted SBP‐box gene, *Colourless nonripening* (*Cnr*), controls fruit ripening in tomato (Manning *et al.*, 2006; Zhong *et al.*, 2013). Thus, modifying the expression patterns of *SPL* genes may lead to crop improvements (Wang and Zhang, 2017).

MicroRNAs (miRNAs) are a class of 20 to 22‐nucleotide RNAs that affect diverse aspects of plant growth and development by promoting either the endonucleolytic cleavage or the translational repression of specifically targeted mRNAs (Cui *et al.*, 2014; Ferreira e Silva *et al.*, 2014; Zhang *et al.*, 2011). MicroRNA156 (miR156) regulates a large network involved in plant growth and development by suppressing the members of the *SPL* gene family, which encode the plant‐specific SBP‐box transcription factors (Ferreira e Silva *et al.*, 2014; Guo *et al.*, 2017; Poethig, 2010; Wang and Wang, 2015; Yu *et al.*, 2015a). In Arabidopsis, miR156 targets 10 members of the *SPL* family (Wu *et al.*, 2009). Similarly, 11 of the 19 SBP‐box (*SPL*) genes are targets of miR156 in rice (Xie *et al.*, 2006). In tomato, 7 of the 17 *SPL* genes are targets of miR156 (Ferreira e Silva *et al.*, 2014; Zhang *et al.*, 2011). In rice, several miR156‐targeted *SPL* genes are associated with yield, including *OsSPL13*, *OsSPL14* and *OsSPL16* (Jiao *et al.*, 2010; Miura *et al.*, 2010; Si *et al.*, 2016; Wang *et al.*, 2015; Wang *et al.*, 2012). In bread wheat, *TaSPL3* and *TaSPL17* are miR156‐targeted genes involved in the regulation of plant architecture (Liu *et al.*, 2017). In Arabidopsis, *AtSPL3*, *AtSPL4* and *AtSPL5* are closely related and miR156‐targeted genes that promote shoot development and flowering (Gandikota *et al.*, 2007; Wu and Poethig, 2006; Xu *et al.*, 2016). Thus, miR156‐SPL modules coordinately regulate plant growth and development traits in crops.

The FT protein encoded by *FLOWERING LOCUS T* (*FT*) is a key component at the convergence of several signalling pathways that serves as a signal for the initiation of flowering (i.e., florigen) in Arabidopsis (Hayama *et al.*, 2003; Kardailsky *et al.*, 1999; Kobayashi *et al.*, 1999; Samach *et al.*, 2000). Its function as a floral inducer has been demonstrated in several plant species (Hecht *et al.*, 2005; Wang *et al.*, 2014). *SINGLE‐FLOWER TRUSS* (*SFT*), the tomato ortholog of *FT*, regulates primary flowering time, sympodial habit and flower morphology (Lifschitz *et al.*, 2006). Similar to the Arabidopsis *FT−/−* (*ft/ft*) mutants, the tomato *SFT−/−* (*sft/sft*) plants flower later than wild type. Moreover, only a few inflorescences develop before they revert to indeterminate vegetative branches that infrequently produce single fertile flowers (Krieger *et al.*, 2010). Significantly, the levels of *SFT* transcripts dramatically decrease in 35S‐miR156a transgenic tomato plants that resemble the *sft* mutant (Zhang *et al.*, 2011). However, the molecular mechanism that links miR156a to the expression of *SFT* in tomato remains unknown.

Our previously research demonstrated that microRNA156a (miR156a) targets seven *SPL* genes and regulates fruit size, fruit yield and the development of both the vegetative inflorescence and the lateral branches in tomato (Zhang *et al.*, 2011). To determine the molecular mechanism responsible for the phenotypes induced by 35S‐miR156a, we conducted a functional analysis of miR156a targeted genes using a transgenic approach. We show that the suppression of *SPL13* in transgenic tomato lines increased the number of vegetative inflorescences and lateral branches, decreased the flower and fruit number and reduced the size of fruits. Almost all of these phenotypes were also observed in transgenic tomato lines harbouring a 35S‐miR156a transgene. Based on these results, we thought that *SPL13* could be the major target of miR156a that regulates inflorescence morphogenesis and lateral branch development in tomato. Significantly, we found that SPL13 affects fruit yield in tomato by directly targeting *SFT*—a major regulator of flowering. Our findings provide new insight into the miR156a‐SlSPL‐based mechanism that regulates tomato yield and plant architecture.

## Results

### RNAi suppression of *SPL13* and overexpression of miR156a produce similar phenotypes

**Figure 1:**
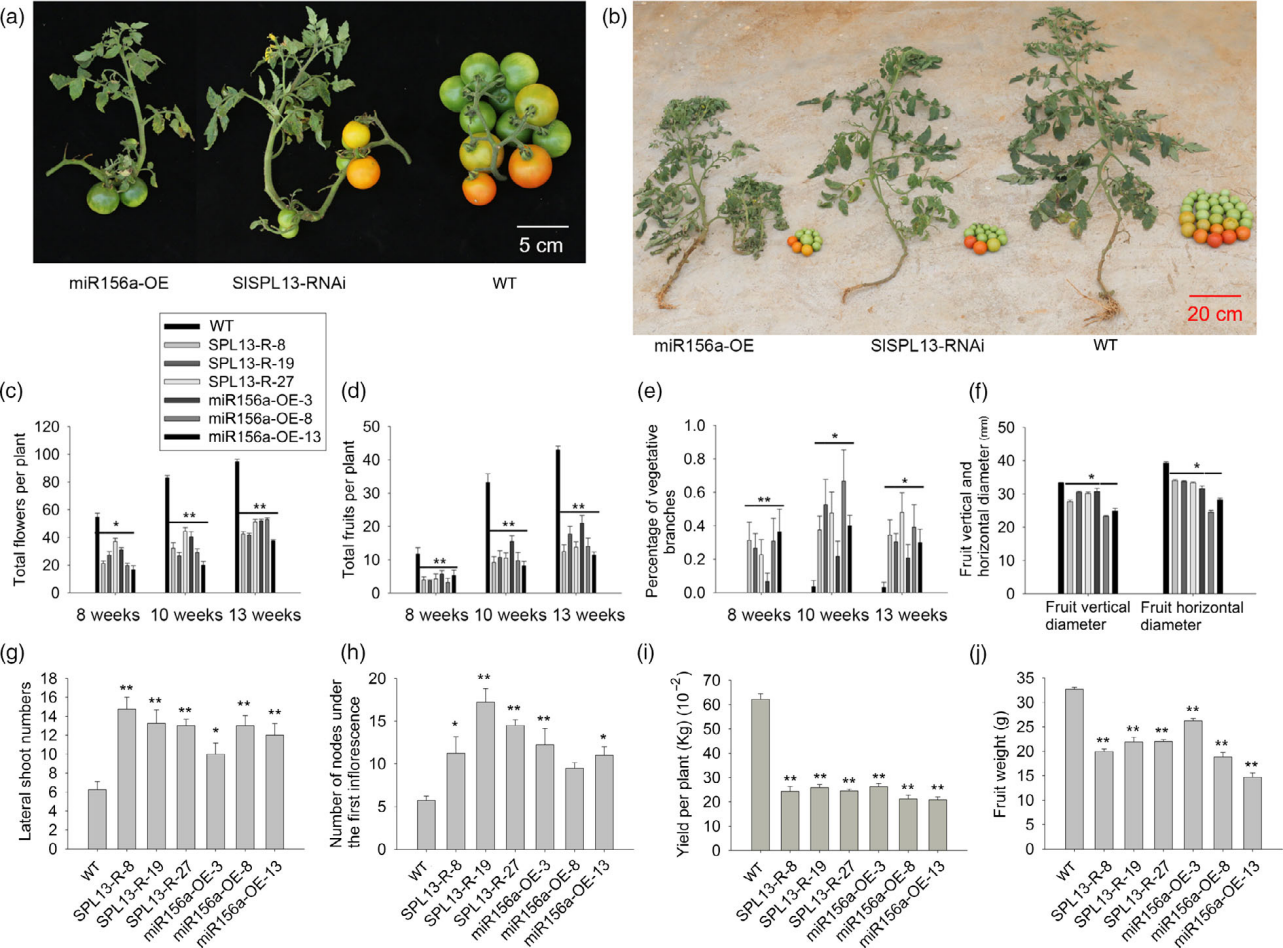
Transgenic *SPL13‐*RNAi and 35S‐miR156a tomato plants are phenotypically similar. (a) Second inflorescence from representative transgenic (left and middle) and WT (right) tomato plants at 10 weeks after planting. (b) Total fruit yield per plant for representative transgenic (left and middle) and WT (right) tomato plants. All transgenic lines are in the Ailsa Craig background. (c–e) Accumulation of flowers, fruits and the percentage of vegetative branch inflorescences per plant at three developmental stages. (f) Statistical comparison of the vertical and horizontal diameters of the fruit from the transgenic and WT tomato plants. (g, h) Lateral branch number and number of nodes from the first inflorescence in the transgenic and WT tomato plants at seven weeks after planting. (i, j) Mean values for the total fruit yield and fruit weight from the transgenic and WT tomato plants. Three transgenic lines from four representative transgenic plants and four representative WT plants were selected for statistical comparisons. Asterisks indicate statistically significant differences relative to the wild type and were determined using *t*‐tests. *, *P* < 0.05, **, *P* < 0.01.

miR156a plays vital roles in tomato development and reproduction. In tomato, miR156a targets 7 of the 17 genes that encode SBP‐box proteins, including *SlSPL2*, *CNR*, *SlSPL3*, *SlSPL6a*, *SlSPL6b*, *SlSPL15* and *SPL13* (Zhang *et al.*, 2011). To determine the molecular mechanism used by miR156a to regulate development and reproduction in tomato, all seven target SPL genes were functionally characterized using a transgenic approach. We found that the suppression of the *SPL13* gene by RNA interference (RNAi) and the overexpression of miR156a in tomato plants produced similar phenotypes (Figure 1, Figure S1a).

Seven weeks after germination, the number of lateral branches produced by the *SPL13‐*RNAi and 35S‐miR156a lines was about twofold more than the wild‐type (WT) plants (Ailsa Craig) (Figure 1g). Eight weeks after germination, the number of sympodial units under the first inflorescence in the *SPL13‐*RNAi and 35S‐miR156a plants was two‐ to threefold more than in the WT plants (Figure 1h), which was also correlated with fewer flowers and reduced yield (Figure 1c,d,i,j). Moreover, the inflorescences of the *SPL13‐*RNAi and 35S‐miR156a plants reverted to indeterminate vegetative branches, and their fruits were scattered on their branches (Figure 1a). We also examined the numbers of flowers and fruits through the entire growing season. The transgenic plants produced 20–53 flowers and 4–21 fruits. In contrast, WT produced 55–95 flowers and 12–43 fruits (Figure 1b‐d). The percentage of vegetative branches was examined in both transgenic and WT tomato plants. In WT, <5% of the shoots were definitively vegetative inflorescence shoots. In contrast, approximately 40% of the additional shoots generated in the transgenic plants were vegetative inflorescence shoots (Figure 1e). As a result, in the transgenic plants, we observed an approximately 40% reduction in the total yield and a 45%–70% reduction in the average fruit weight relative to the WT plants (Figure 1i,j). The vertical and horizontal diameters of the fruit were also reduced in the transgenic plants relative to the WT (Figure 1f). Thus, the *SPL13*‐RNAi plants and the 35S‐miR156a plants are phenotypically similar.

### CR‐*spl13* is phenotypically similar to the 35S‐miR156a and *SPL13‐*RNAi plants

**Figure 2:**
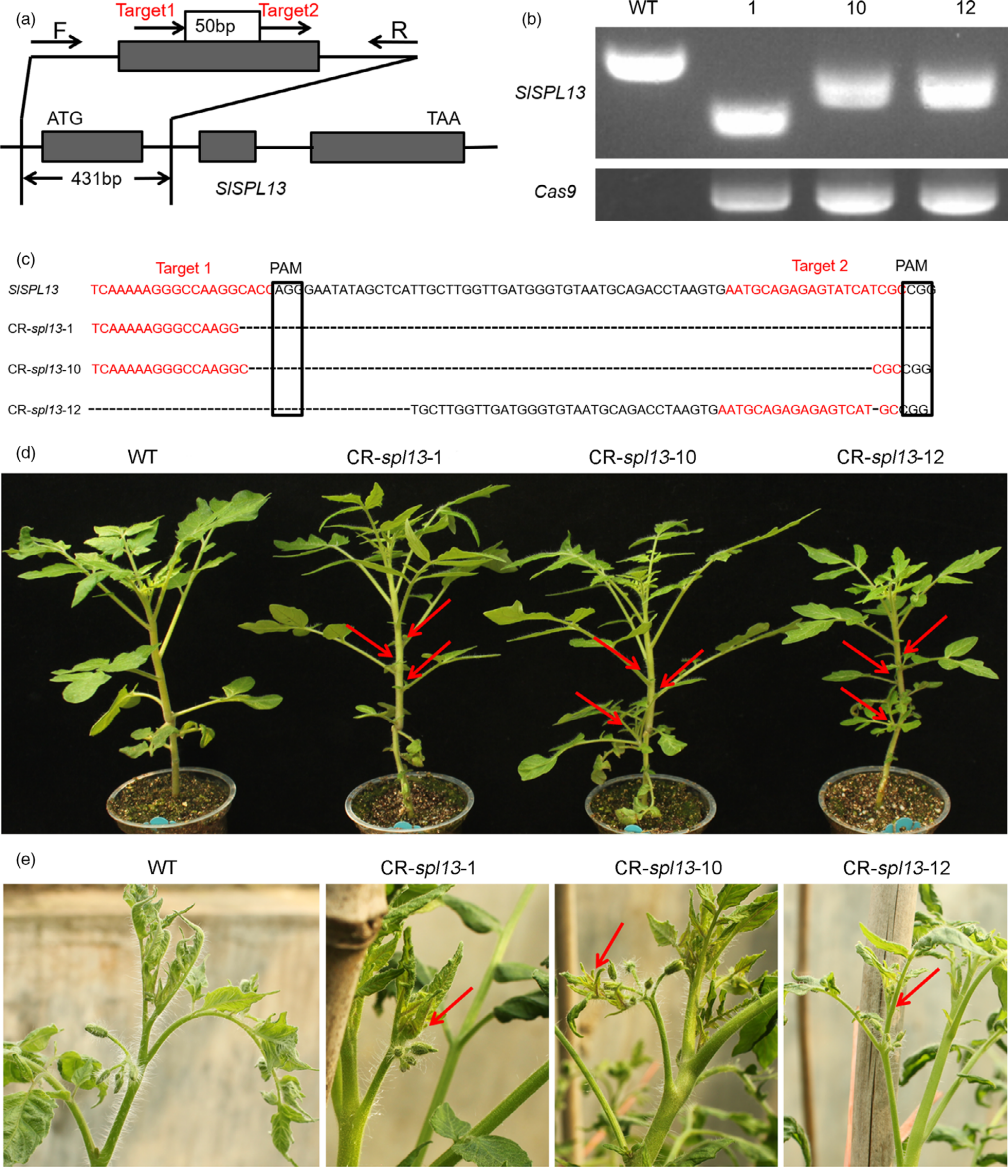
Increased vegetative branches from inflorescences and lateral branches in the CR‐*spl13* mutants. (a) Schematic illustration of the two sgRNA target sites (red arrows) in *SPL13*. Black arrows represent the location of the primers that were used for PCR‐based genotyping. (b) PCR‐based analysis of three CR‐*spl13* mutant alleles with different amplicon lengths. Amplification of the *Cas9* transgene is shown as a positive control. (c) Verification of the CR‐*spl13* mutant alleles by DNA sequencing analysis. We found a deletion that spanned the two sgRNA target sites in all three lines. The red font indicates sgRNA target sequences. The black boxes indicate protospacer‐adjacent motif (PAM) sequences. (d, e) Lateral branch and inflorescence vegetative branch phenotypes in the *SPL13* CRISPR/Cas9 lines. Red arrows indicate lateral branches in the leaf axil. (d) Vegetative branches in the inflorescence (e). WT (AC) was used as the control.

To further evaluate the correlation between the phenotypes caused by knocking down the expression of the *SPL13* gene and by the overexpression of miR156a, we used CRISPR‐Cas9 to generate transgenic tomato lines (CR*‐spl13*) containing knockout alleles of the *SPL13* gene (Figure 2a‐c). We observed long deletions and single‐base mutations in the CR‐*spl13* lines 1, 10 and 12 (Fig. 2b‐c). Using a PCR‐based genotyping procedure, we identified an *spl13* knockout mutant with a long deletion in the *SPL13* gene and lacking the CRISPR‐Cas9‐containing transgene in the T1 generation (Figure S2a, b). We found that the number of vegetative branches that developed from inflorescences increased and that the lateral branches initiated earlier in these CRISPR‐Cas9‐induced *spl13* mutants than in WT plants (Figure 2d‐e, Figure S1f‐g, S2c–f). Phenotypic and statistical analyses showed that flower and fruit number, percentage of vegetative branches, fruit size and yield in the CR‐*spl13* lines without the CRISPR‐Cas9‐containing transgene were similar to the *SPL13‐*RNAi lines (Figure 1, Figure S2g‐m). Our results demonstrate that the CR*‐spl13* transgenic tomato plants, *SPL13‐*RNAi lines and 35S‐miR156a lines are phenotypically similar. These results suggest that the phenotypes of the *SPL13*‐RNAi transgenic lines are mainly caused by down‐regulating the expression of *SPL13* and that the main target gene of miR156a in the 35S‐miR156a lines is *SPL13*.

### miR156a interferes with the expression of *SPL13*

**Figure 3:**
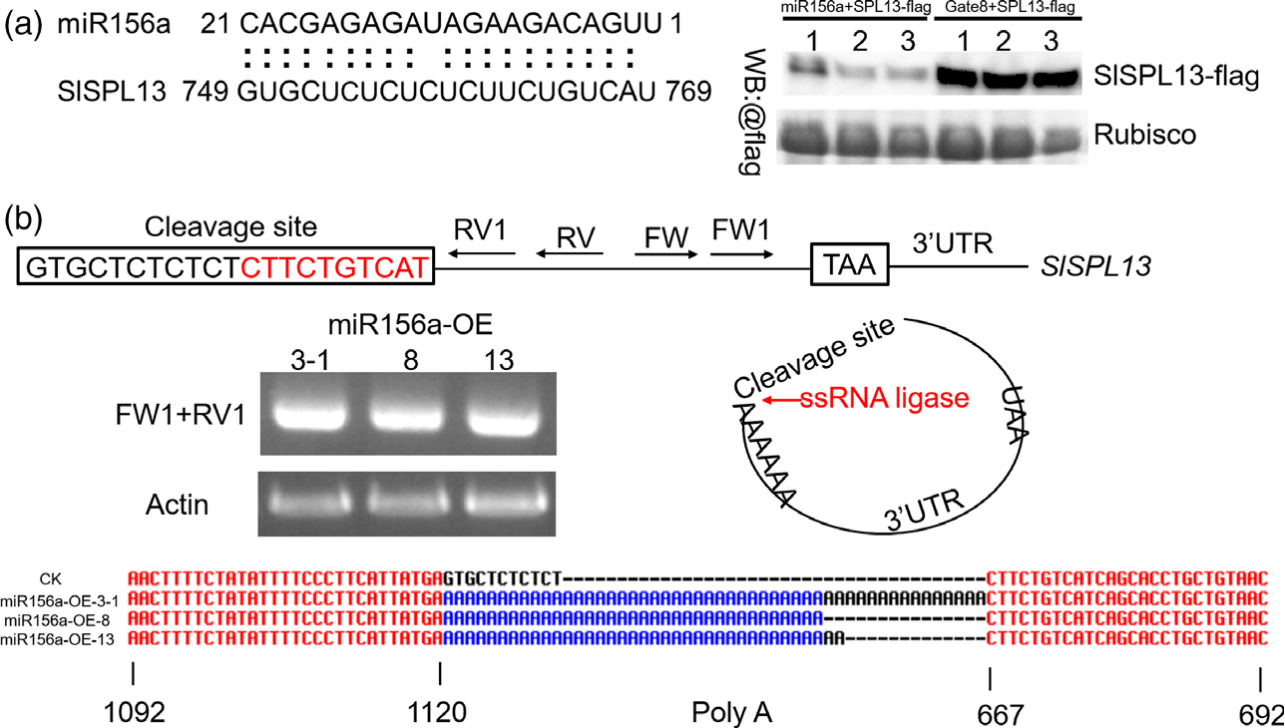
Targeting of SPL13 by miR156a *in vivo*. (a) FLAG‐tagged SPL13 and 35S‐miR156a constructs transiently co‐expressed in the leaves of *N. benthamiana*. An alignment of the target DNA sequences are shown (left panel). FLAG‐tagged SPL13 and the empty vector (pHELLSGATE8) were transiently co‐expressed as controls. Leaf extracts were analysed by Western blotting (WB) using anti‐FLAG antibodies. These experiments were repeated three times and yielded similar results each time. (b) Model for 5'race in the 35S‐miR156a plants. Four 35S‐miR156a lines were used for these experiments. The cleaved RNA was self‐ligated using ssRNA ligase (middle right) and then reverse transcribed. Nested PCR was performed using the indicated primers (top). The product was visible after the second reaction (middle left). The products were ligated into the vector. The sequences detected using the M13 primer are shown (bottom).

**Figure 4:**
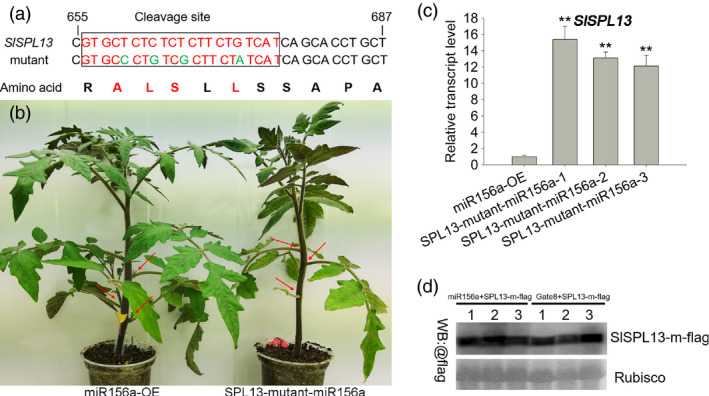
Phenotypic characterization of plants overexpressing a site‐directed mutant allele of *SPL13* (pCAMBIA 1302, hygromycin resistance) in the 35S‐miR156a background. (a) Four nucleotides were changed in the cleavage site of the *SPL13*. These mutations prevent cleavage but do not change the amino acid sequence. This mutant allele of *SPL13* was amplified using nested PCR. (b) Lateral branch phenotype in the *SPL13* mutant and miR156a lines. Red arrows indicate the leaf axils. (c) Quantitative PCR analysis of *SPL13* expression in young leaves from three transgenic and miR156a lines. Asterisks indicate statistically significant differences relative to the miR156a‐OE and were determined using *t*‐tests. **, *P* < 0.01. (d) FLAG‐tagged *SPL13* mutant and 35S‐miR156a constructs transiently co‐expressed in the leaves of *N. benthamiana*. The FLAG‐tagged SPL13 mutant (SPL13‐m‐flag) and the empty vector (pHELLSGATE8) were transiently co‐expressed. Leaf extracts were analysed by Western blotting (WB) using anti‐FLAG antibodies. These experiments were repeated three times and yielded similar results each time.

MicroRNAs (miRNAs) regulate gene expression by directing endonucleolytic cleavage or by inhibiting the translation of target mRNAs (Jones‐Rhoades *et al.*, 2006). Previously, we determined that the transcript levels of the *SPL13* gene were significantly decreased in the 35S‐miR156a transgenic plants (Zhang *et al.*, 2011). To further test whether the *SPL13* gene is the target of miR156a, we quantified the levels of the epitope‐tagged SPL13 (SPL13‐FLAG) protein when it was co‐expressed with miR156a in the heterologous *N. benthamiana* transient expression system. We found that SPL13‐FLAG accumulated to high levels when it was co‐expression with the empty vector and that the SPL13‐FLAG protein accumulated to significantly lower levels when the empty vector was replaced with a vector containing the 35S‐miR156a transgene (Figure 3a, Figure S3c). We also found that when SPL13‐m‐FLAG was co‐expressed with miR156a in the heterologous *N. benthamiana* transient expression system, the levels of the SPL13‐FLAG protein were not significantly different relative to the empty vector (Figure 4a, d, Figure S3d). These data indicate that miR156a can affect the accumulation of the SPL13 protein. We used a 5′ RACE assay to directly confirm that the mRNA encoding SPL13 is the target of miR156a. After two steps of nested PCR, a single band was obtained. The sequence of this PCR product indicates that the cleaved *SPL13* RNA was self‐ligated to the poly(A) tail. These data also indicate that the predicted cleavage site and the actual cleavage site are exactly the same (Figure 3b, Figure S3a–b). A functional complementation assay was designed to test whether *SPL13* is the target of miR156a. We found that when a transgene containing a mutant allele of SPL13 that contains four mutations in the miR156a cleavage site and encodes an SLP13 protein with a wild‐type amino acid sequence was overexpressed in the 35S‐miR156a background, there were fewer lateral branches relative to the 35S‐miR156a line (Figure 4a–c). Indeed, even the apex disappeared in the later stages of growth. Based on these observations, we conclude that the overexpression of *SPL13* caused the lateral branches of the 35S‐miR156a plants to revert. These results indicate that overexpressing miR156a attenuates the accumulation of the SPL13 protein by interfering with the accumulation of the *SPL13* mRNA.

### SPL13 is a TF

**Figure 5:**
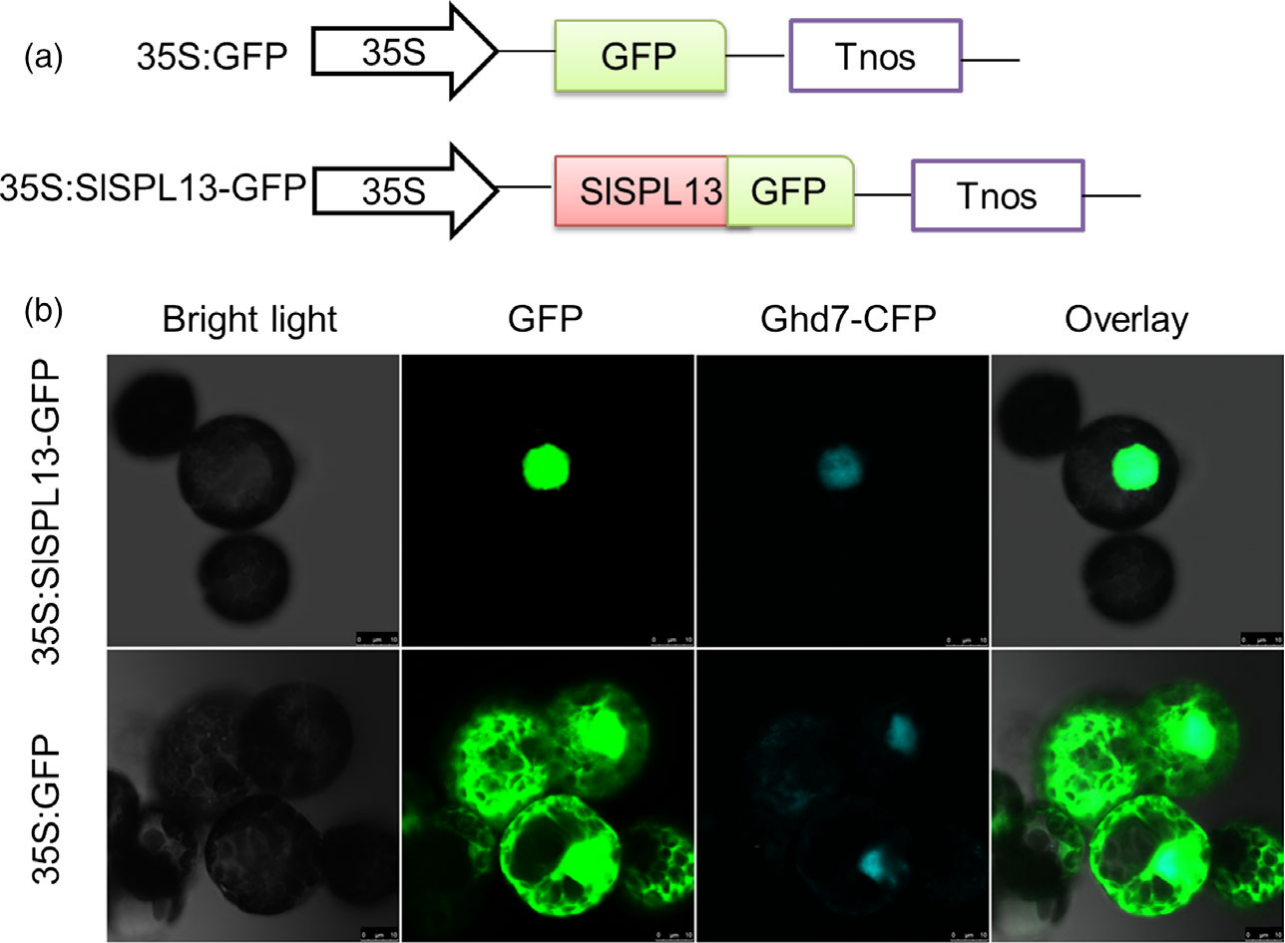
Subcellular localization of SPL13. (a) Schematic diagrams of the constructs used to determine subcellular localization. The *SPL13* CDS without the stop codon was amplified by PCR and fused to the 5′ end of the open reading frame encoding GFP in pCAMBIA 1302. The expression of SPL13‐GFP was driven by the CaMV 35S. (b) Transient expression of 35S:SPL13‐GFP and 35S:GFP in tobacco (*N. benthamiana*) protoplasts. The nuclei were identified by co‐expressing the nuclear marker Ghd7‐CFP with both 35S:SPL13‐GFP and 35S:GFP. Fluorescence images were acquired using a confocal laser scanning microscope (Leica TCS SP2, MRC Centre for Regenerative Medicine, The University of Edinburgh, Edinburgh, UK) after incubating the protoplasts at 28 °C for 12–16 h. Representative micrographs are shown. Bars, 10 μm.

Because a bioinformatics analysis indicated that the SBP domain of the SPL13 protein contains a nuclear localization signal, we next determined the subcellular distribution of SPL13 fused to a green fluorescence protein (GFP) in *N. benthamiana* protoplasts using confocal laser scanning microscopy. We included the nuclear marker protein Ghd7（Grain number, plant height and heading date7）fused with the cyan fluorescent protein (CFP) in our assay to unambiguously identify the nucleus. In these assays, the SPL13‐GFP and Ghd7‐CFP fusion proteins were co‐expressed in the *N. benthamiana* protoplasts. We found that the SPL13‐GFP protein was exclusively localized to the nucleus, based on the complete overlap of the fluorescence emitted by SPL13‐GFP and Ghd7‐CFP (Figure 5). In contrast, fluorescence emitted from the free GFP was distributed throughout the cell (Figure 5). Based on these observations, we conclude that SPL13 accumulates in the nucleus, where it may function as a transcription factor.

### SPL13 promotes the expression of the flowering gene *SFT*

In the tomato *sft* mutant, flowering is delayed and the inflorescences revert to indeterminate vegetative branches or become single fertile flowers. Thus, the tomato *sft* mutant has fewer flowers and a lower fruit yield than WT (Krieger *et al.*, 2010). The *SPL13*‐RNAi transgenic tomato plants and the *sft* mutant are phenotypically similar. Their flowering is delayed, the number of their vegetative inflorescence shoots is increased by approximately 40% (Figure 1a,e,h), they develop fewer flowers and their fruit yield is reduced (Figure 1b,d,i). In contrast, the overexpression of *SFT* in tomato induces the development of simple leaves, short internodes and early flowering (Lifschitz *et al.*, 2006). Moreover, in addition to simple leaves we found reduced numbers of lateral branches in our *SPL13* overexpressing tomato lines (Figure S1c,d,f). Additionally, our real‐time RT‐PCR analysis indicated that the *SFT* transcripts were significantly lower in the CR*‐spl13* lines but higher in the 35S‐SPL13 lines relative to the WT plants (Figures S1e, S9a). Thus, the miR156a‐targeted *SPL13* gene appears to serve as the core transcription factor that regulates the expression of the *SFT* gene.

### SPL13 positively regulates *SFT* expression by directly binding regulatory *cis*‐elements in the *SFT* promoter

**Figure 6:**
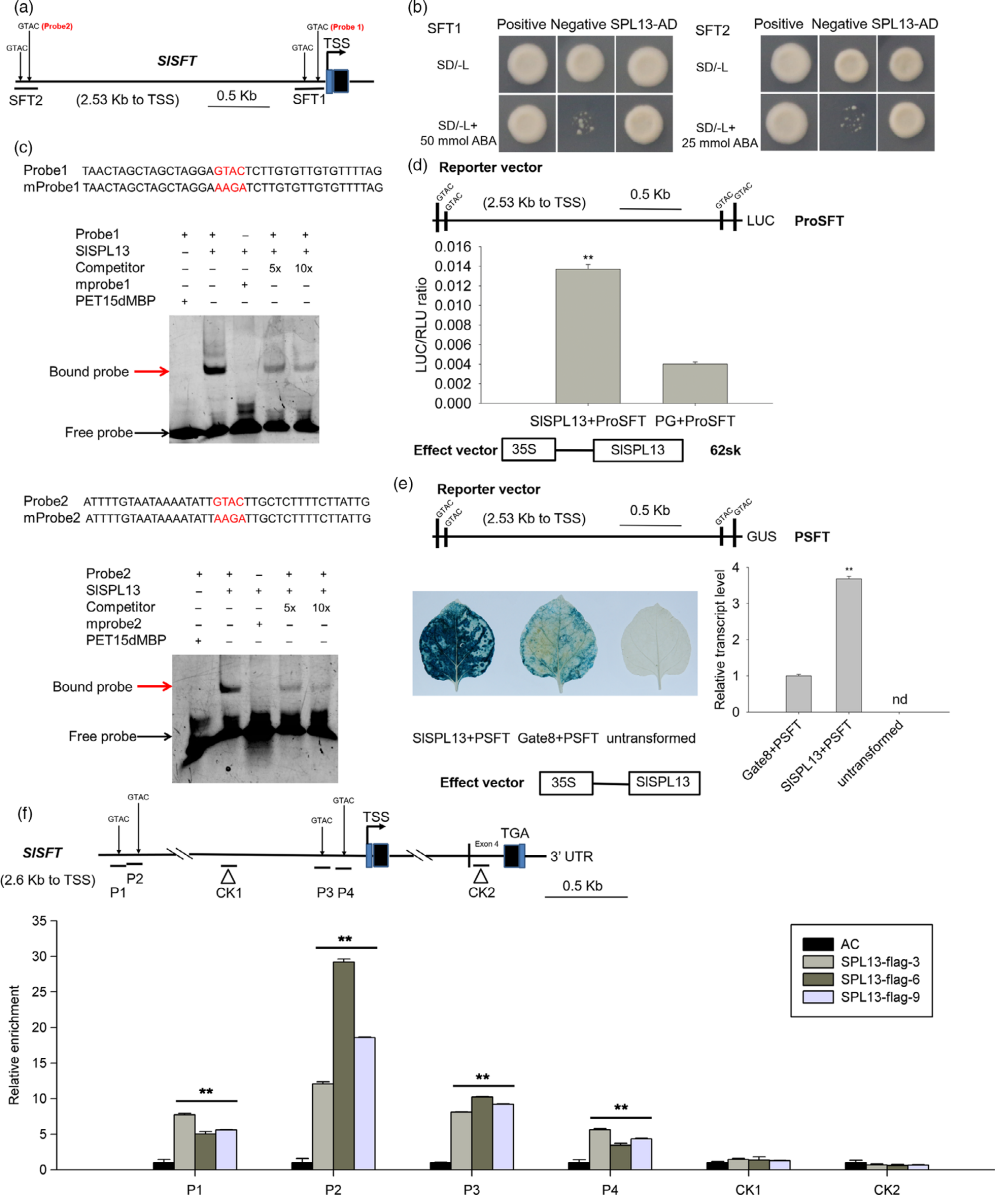
SPL13 binds to the *SFT* promoter and activates *SFT* expression. (a) Schematic diagram of the 2533 bp *SFT* promoter region. Four GTAC‐containing *cis*‐elements were identified in the promoter of *SFT*. Two constructs containing two different promoter fragments (SFT1 and SFT2) were used in the yeast one‐hybrid (Y1H) assay. SFT2 and SFT1 contain from −2528 to −2246 bp and from −289 to 0 relative to the translational start codon, respectively. (b) Y1H analysis of SPL13 binding to the different core sequences from the *SFT* promoter. The bait vectors, SFT1 and SFT2, and the SPL13‐containing prey vector were introduced into the yeast strain Y1H gold. The enhanced resistance to ABA indicates an interaction between the bait and prey. Co‐transformation of the bait vectors, SFT1 and SFT2, with either pGADT7 or pGADT‐Rec2‐53 served as negative and positive controls, respectively. (c) EMSA assay testing the binding of SPL13 to *SFT* promoter fragments. Two 39‐bp single‐strand oligonucleotide probes containing GTAC sequences were synthesized and labelled with biotin. Unlabelled fragments were used as negative controls. The His‐6‐MBP‐SPL13 protein was incubated with the biotin‐labelled probe containing GTAC or the mutated probe (mprobe) containing the AAGA sequence. The unlabelled fragment was used as a competitor. + and ‐ indicate the presence and absence of the corresponding probe or protein. The arrows indicate the protein–DNA complex (red arrow) or free probe (black arrow). (d) Dual luciferase system analysis of SPL13 binding the promoter of *SFT*. The *SFT* promoter fragment was inserted into the reporter vector (pGreen II 0800 LUC). *SPL13* was inserted into the effector vector (pGreen II 62‐SK). The resulting constructs were transiently expressed in tobacco (*Nicotiana benthamiana*) leaves by *Agrobacterium tumefaciens*‐mediated transformation. LUC, firefly luciferase activity; RLU, Renilla luciferase activity; PG, the empty vector of pGreen II 62‐SK. The *SFT* promoter plus PG was used as a control. Values are presented as means ± SE (*n* = 3). The asterisks indicate statistically significant differences that were determined using the *t*‐test: *, *P* < 0.05, **, *P* < 0.01. (e) GAL4/UAS‐based analysis on SPL13 binding to the *SFT* promoter. The promoter of *SFT* was fused to an open reading frame encoding the GUS protein (PSFT‐GUS), and SPL13 was expressed in the pHELLSGATE8 vector (35S‐SPL13). The resulting constructs were transiently co‐expressed in the leaves of *N. benthamiana*. PSFT‐GUS and the empty vector pHELLSGATE8 were included as controls. Values are presented as means ± SE (*n* = 3). The asterisks indicate statistically significant differences that were determined using the *t*‐test. *, *P* < 0.05, **, *P* < 0.01. nd, Not detected. (f) ChIP qPCR analysis of SPL13 binding to the *SFT* promoter in the WT and 35S‐SPL13‐flag transgenic tomato plants. The P1, P2, P3 and P4 fragments contain GTAC *cis*‐elements. CK1 and CK2 are located in the promoter and coding sequence of *SFT*, respectively. The relative enrichment of the six promoter fragments in the young leaves of three transgenic lines was quantified using qPCR. Data were normalized to those of the WT plants. This experiment was repeated three times. The data presented are the means ± SE. The asterisks indicate statistically significant differences relative to the WT that were determined using the *t*‐test: *, *P* < 0.05, **, *P* < 0.01.

The SPL transcription factors regulate the transcription of target genes by binding GTAC‐containing *cis*‐elements (Usami *et al.*, 2009; Yu *et al.*, 2010). Thus, to test whether SPL13 might directly bind the *SFT* promoter and regulate the transcription of the *SFT* gene, we searched for potential GTAC‐containing *cis*‐elements in the *SFT* promoter. We found four GTAC sequences (Figure 6a). To further determine the relative importance of these four GTAC sequences, two reporter constructs containing two different *SFT* promoter fragments from −2528 to −2246 bp (hereafter referred to as SFT2) and from −289 to −0 bp (hereafter referred to as SFT1) relative to the translational start codon of the *SFT* gene (Figure 6a) were tested in Y1H assays. Our result indicates that SPL13 can bind both SFT1 and SFT2 (Figure 6b).

The binding of SPL13 to the GTAC *cis*‐elements was further analysed using the electrophoretic mobility shift assay (EMSA) with a recombinant SPL13 protein expressed and purified from *E. coli* (Figure S4). Biotin‐labelled oligonucleotide probes containing the core GTAC sequences from SFT1 and SFT2 were generated, and the same unlabelled oligonucleotides were used as competitors (Figure 6c). The SPL13 protein was incubated with the biotin‐labelled probes to generate protein–DNA complexes that were detected based on their slower mobilities in EMSA assays. The protein–DNA complexes were significantly reduced when we added unlabelled competitor probes. In addition, the protein–DNA complex was not detected when the core GTAC sequence of the *cis*‐element was mutated to AAGA (Figure 6c). Additionally, promoter fragments from *SFT* (P1, P2, P3 and P4) that contain the GTAC sequences were enriched by SPL13‐FLAG in ChIP‐PCR experiments. In contrast, the promoter fragments from *SFT* lacking the GTAC sequence (CK1 and CK2) were not enriched by SPL13‐FLAG (Figures 6f, S5). Based on these data, we conclude that the FLAG‐tagged SPL13 protein can bind the core GTAC motif in the *SFT* promoter. Taken together, our results support the idea that SPL13 directly binds GTAC‐containing *cis*‐elements in the *SFT* promoter.

To test whether SPL13 can directly regulate the expression of the *SFT* gene, the *SFT* promoter was cloned into the luciferase‐based reporter vector (pGreen II 0800 LUC) and SPL13 was cloned into the effector vector (pGreen II 62‐SK) to generate an artificial reporter gene system. The LUC/RLU ratio assay revealed that SPL13 can activate the *SFT* promoter‐driven *Luc* reporter gene (Figure 6d). This result was independently verified with an *SFT* promoter‐driven GUS reporter gene (Figure 6e). Together, these results indicate that SPL13 is a transcriptional activator of *SFT in vivo*.

## Discussion

### SPL13 is a key factor in miR156a‐regulated tomato plant development

The effect of miR156 on yield and plant architecture has been studied in rice, bread wheat, maize, Arabidopsis and tomato (Chuck *et al.*, 2007; Liu *et al.*, 2017; Luo *et al.*, 2012; Schwab *et al.*, 2005; Xie *et al.*, 2012; Zhang *et al.*, 2011). In angiosperms, the lateral meristems mainly determine the overall morphology and reproductive capability of the plant (Martin‐Trillo *et al.*, 2011; Otsuga *et al.*, 2001). The *SPL* genes encode plant‐specific transcription factors that are targeted by miR156 and participate in the regulation of multiple developmental processes (Ferreira e Silva *et al.*, 2014; Zhang *et al.*, 2011). In rice, miR156‐targeted *OsSPL13* is associated with yield (Si *et al.*, 2016). miR156‐targeted *MsSPL13* regulates vegetative and reproductive development in alfalfa (Gao *et al.*, 2016; Gao *et al.*, 2018). Nonetheless, there are few studies on miR156‐targeted *SPL13* in tomato. Thus, it appeared important to determine the biological function of the *SlSPL13* gene and the influence of miR156a on *SlSPL13* expression in tomato.

In tomato, miR156b and its targets—mRNAs encoding SPLs—can regulate fleshy fruit and axillary shoot development in cv. Micro‐Tom (Ferreira e Silva *et al.*, 2014). Our previously published work demonstrated that miR156a targets the *SPLs* that regulate fruit size, fruit yield and the development of both the vegetative inflorescence and lateral branches in cv. Ailsa Craig (Zhang *et al.*, 2011). In both the miR156a and miR156b overexpression lines, flowering time was delayed, and the number of fruits was reduced (Ferreira e Silva *et al.*, 2014; Zhang *et al.*, 2011), but the ovary and inflorescence morphogenesis were different. These differences might be due to the different backgrounds and targets. Recent studies revealed that the integration of miR156b‐targeted *SPLs* (*SPL3* and *SPL15*), GA and the miR319‐targeted *LANCEOLATE* (*LA*) control flower initiation in tomato by up‐regulating the expression of the *SFT* gene in leaves (Silva *et al.*, 2019). To test whether transgenic tomato plants suppressing either *SPL3* or *SPL15* and plants overexpressing miR156a are phenotypically similar, we down‐regulated the expression of *SPL3* and *SPL15* and observed no obvious phenotypic difference relative to wild type. In this study, we report that down‐regulating the expression of *SPL13* in tomato leads to late‐flowering and vegetative inflorescence phenotypes (Figure 1a,e,h). Consistent with this observation, expression profiling revealed that *SPL13* is mainly expressed in leaves and flowers (Figure S1b)*.* Moreover, in the 35S‐miR156a lines, in contrast to wild type, the lateral branches developed very early and almost every leaf axil formed a lateral branch. In contrast, when we overexpressed *SPL13* in the 35S‐miR156a background, the transgenic plants had less lateral branches relative to the 35S‐miR156a control (Figure 4a–c). Based on these results, we conclude that miR156a regulates inflorescence morphogenesis and lateral branch development in tomato by targeting *SPL13*.

Amino acid sequence analysis provides more evidence that among the seven *SPL* genes that are targeted by miR156a in tomato the *SPL13* gene is distinct. In a phylogenetic analysis, SPL13 formed a separate branch relative to these other SPL proteins (Figure S6). Moreover, an amino acid sequence alignment of the seven proteins encoded by miR156a‐targeted mRNAs in tomato revealed that SPL13 has a distinct Q to L substitution in the SBP‐box domain, which may affect the activity of SPL13 (Figure S7). Based on these data, we conclude that SPL13 may be a core transcription factor that regulates growth and development in tomato. Consistent with this idea, a qRT‐PCR analysis indicated that the expression of *SPL2*, *CNR*, *SPL6a*, *SPL6b* was down‐regulated in the CR*‐spl13* lines and that the expression of *SPL3* and *SPL15* was not significantly different (Figure S8). The *SPL* gene *UB3* (*UNBRANCHED3*) has been reported to bind the promoter of *OsSPL14* and *OsSPL17* in rice (Du *et al.*, 2017, 2017). Thus, we hypothesize that similarly, SPL13 may regulate growth and development in tomato by affecting the expression of miR156a‐targeted genes (*SPL2*, *CNR*, *SPL6a*, *SPL6b*).

### miR156a‐targeted *SPL13* regulates inflorescence morphogenesis through the SFT/FT‐AP1 pathway

Forward genetic approaches in tomato have yielded many genes associated with inflorescence development that influence yield and fruit harvest (Emmanuel and Levy, 2002; Perilleux *et al.*, 2014). For instance, in the late‐flowering mutants *falsiflora* (*fa*) and *single flower truss* (*sft*), the inflorescence shows an increased propensity to produce vegetative tissue. Indeed, leaf production resumes in the inflorescence (Allen and Sussex, 1996; Molinero‐Rosales *et al.*, 1999; Molinero‐Rosales *et al.*, 2004; Perilleux *et al.*, 2014). The *terminating flower* (*tmf*) mutant has an early flowering phenotype (MacAlister *et al.*, 2012). *ANANTHA* (*AN*) regulates inflorescence architecture by controlling the early stages of floral meristem development (Allen and Sussex, 1996; Lippman *et al.*, 2008). Vegetative growth was resumed after a few flowers were formed in the *jointless* mutant, which is similar to the *sft* mutant (Mao *et al.*, 2000; Quinet *et al.*, 2006; Szymkowiak and Irish, 1999). We tested the expression of these genes in the CR‐*spl13* lines using qRT‐PCR. We found that the expression of *SFT* and *FA* was significantly down‐regulated and that the expression of *JOINTLESS*, *TMF* and *AN* was not significantly affected (Figures S9a, b, S9b, d).

FT serves as a signal to initiate flowering in Arabidopsis and other plant species (Du *et al.*, 2017, 2017; Hecht *et al.*, 2005; Samach *et al.*, 2000; Wang *et al.*, 2014). Mutations in *SFT*, the tomato ortholog of *FT*, reduce flower numbers and fruit yield (Krieger *et al.*, 2010; Lifschitz *et al.*, 2006). The inflorescence structures of the 35S‐miR156a tomato plants and the *sft* mutants are indistinguishable. Additionally, the expression of the *SFT* gene was significantly down‐regulated in the 35S‐miR156a tomato plants (Zhang *et al.*, 2011). In this study, we found that the expression of *SFT* was also significantly reduced in the *CR‐spl13* transgenic lines but up‐regulated in *SPL13* overexpressing transgenic lines (Figures S1e, S9a,). Using yeast one‐hybrid assays, transient expression assays in planta, EMSAs and ChIP assays, we demonstrated that SPL13 positively regulates the expression of *SFT*, presumably by directly binding the promoter of *SFT* (Figure 6). *SFT* and *FA* act in parallel pathways as demonstrated by the additive late‐flowering phenotypes of the *fa* and *sft* mutants (Molinero‐Rosales *et al.*, 2004; Perilleux *et al.*, 2014; Thouet *et al.*, 2012). The expression of the *FA* gene was significantly decreased in the CR‐*spl13* tomato plants (Figure S9b). These results imply that SPL13 may affect inflorescence development also by promoting the expression of *FA*.

In tomato, since sympodial shoot development is regulated by the SFT to SP (SELF‐PRUNING) ratio (Jiang *et al.*, 2013; Park *et al.*, 2014; Shalit *et al.*, 2009), the aberrant vegetative inflorescence shoots in the 35S‐miR156a tomato plants may be attributed to the decreased SFT to SP ratio (Zhang *et al.*, 2011). SPL13 positively regulates the expression of *SFT* by directly binding its promoter (Figure 6, Figure S9a). We also examined the possibility of SPL13 binding the promoter of the *SP* gene. We were not able to demonstrate that SPL13 binds the promoter of *SP* (Figure S11). Consistent with this finding, our gene expression assays indicated that the expression of *SFT* and *SP* was down‐regulated and unchanged, respectively, in the CR‐*spl13* plants (Figures S1e, S9a, S10a). This result also illustrates why the ratio of SFT to SP was decreased in the 35S‐miR156a tomato plants and why the decreased *SFT* to *SP* ratio promotes the development of aberrant vegetative inflorescence shoots in CR‐*spl13* and 35S‐miR156a plants.

In *Arabidopsis thaliana*, APETALA1 (AP1) encodes a MADS domain protein, and the flowers of the *ap1* mutant are replaced by shoots or flowers that have shoot‐like characteristics (Wang *et al.*, 2009). In tomato, the *AP1* gene (*SlAP1*) is also a target of the SFT transcription factor (Krieger *et al.*, 2010). The expression of *SlAP1* in the inflorescence shoot apices is strongly reduced in *sft* homozygous mutants (Kobayashi and Weigel, 2007; Krieger *et al.*, 2010). We also found that the expression of *SlAP1* is significantly reduced in CR*‐spl13* transgenic lines (Figure S9c). These results revealed that SPL13 regulates inflorescence development in tomato by affecting the SFT/FT‐AP1 pathway. The *J* (*JOINTLESS*) gene, regulating inflorescence vegetative growth in tomato, encodes a MADS‐box protein. The MADS‐box proteins act in complexes (Mao *et al.*, 2000; Quinet *et al.*, 2006; Szymkowiak and Irish, 1999; Thouet *et al.*, 2012). It was previously reported that in Arabidopsis, J can interact with several MADS‐box proteins such as SUPPRESSOR OF OVEREXPRESSION OF CO1 (SOC1), APETALA1/FRUITFULL (AP1/FUL) and SEPALLATAs (SEPs) in yeast (Leseberg *et al.*, 2008). The expression of the *J* gene was not significantly changed in the CR‐*spl13* tomato plants (Figure S10b). Thus, we speculate that J may regulate inflorescence development by interacting with other MADS‐box proteins. All of these data are consistent with a miR156a‐SPL‐based mechanism regulating inflorescence development in tomato by affecting the SFT/FT‐AP1 pathway. Thus, we hypothesize that the miR156a‐SPL13‐SFT‐AP1 signalling cascade is possibly conserved in diverse crop species.

**Figure 7:**
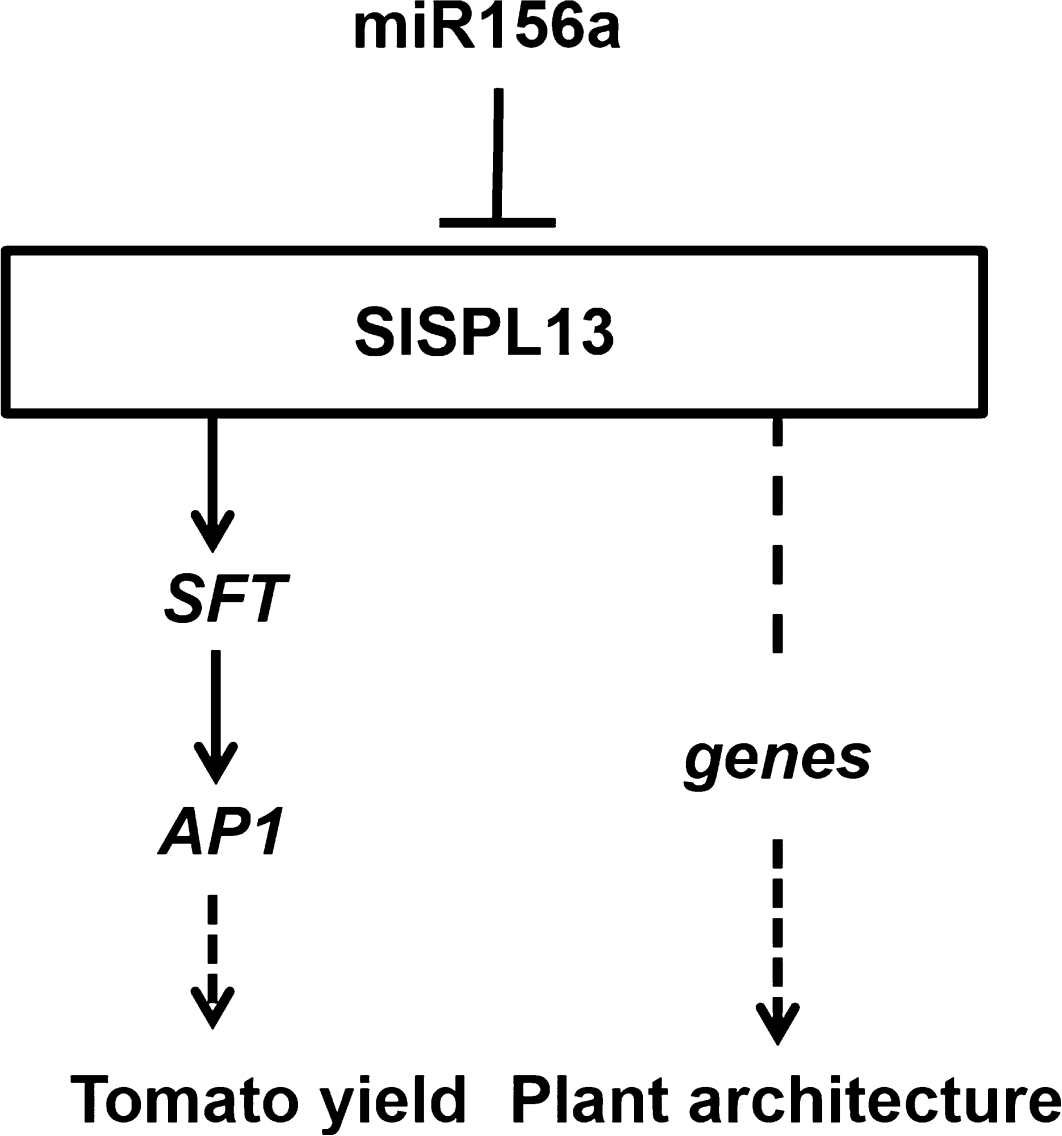
Working model for the regulation of tomato inflorescence development, plant architecture and yield by miR156a‐SPL13.

Based on our findings, we propose a model that includes SPL13 affecting plant yield traits by regulating the expression of *SFT* and the miR156a‐SPL pathway regulating lateral branching. In this model, we propose that at least two distinct pathways exist downstream of SPL13. In one pathway, the miR156a‐regulated SPL13 acts as a transcriptional activator that controls the production of fruit by up‐regulating the expression of target genes, such as *SFT*. In the other pathway, miR156a‐regulated SPL13 might control plant architecture by affecting the expression of vital downstream genes (Figure 7). Thus, manipulation of the miR156a‐SPL pathway has the potential to improve fruit production and plant architecture not only in tomato but also in other crops, studying this pathway in other crops may lead to similar genetic improvements.

## Methods

### Plant materials and growth conditions

The WT tomato cultivar Ailsa Craig (AC) was used for all of the transformation experiments. The WT (AC) and the transgenic lines (T1 generations) were grown in nutrition pots at a greenhouse in Huazhong Agriculture University in Wuhan (30.4°N, 114.2°E), China. *Nicotiana benthamiana* was grown in an environmentally controlled room at 22 °C with a photoperiod that consisted of 16 h of light followed by 8 h of dark.

### RNA isolation and gene expression analysis

Total RNA was extracted from various tissues of the transgenic lines or WT plants using the TRIzol reagent (Invitrogen). Complementary DNAs were synthesized using an M‐MLV reverse transcriptase kit (Toyobo). The LightCycler480 SYBR Green I Master Kit (Roche) was used for the qPCR analysis. Three biological replicates from each genotype were analysed to test for statistically significant differences. The actin gene (BT013524) was used as an internal control. The primer sequences used in real‐time PCR are listed in Table S1.

### Gene constructs and tomato transformations

The full‐length ORF and RNAi fragments for *SPL13* and six other target genes were amplified from tomato cDNA using the KOD‐Plus DNA polymerase (Toyobo). The CRISPR cas9 vector targeted two sites in the first exon of the ORF of SPL13 and was designed at CRISPR‐PLANT (http://www.genome.arizona.edu/crispr/CRISPRsearch.html). The vectors were introduced into the *Agrobacterium tumefaciens* strain C58. These strains were used for tomato Ailsa Craig (AC) transformations as described previously (Jones *et al.*, 2002). Genomic DNA was extracted from transgenic plants using the CTAB method as described by Murray and Thompson (1980). The genomic DNA was analysed using PCR‐based markers to test for transgenic plants. The primers used in these experiments are listed in Table S1.

### Yeast one‐hybrid assay

The Y1H assay was used to test whether SPL13 can interact with the *SFT* promoter. The full‐length *SPL13* ORF sequence was amplified from tomato cDNA and cloned into pGADT7 (Clontech). Two promoter fragments (−2528 to −2246 bp and −289 to −0 relative to the translation initiation codon of the *SFT* gene) were amplified from tomato genomic DNA and cloned into PAbai (Clontech). The primer sequences are provided in Table S1. PAbai was first introduced into the Y1H gold yeast (Clontech) and cultured on SD/‐Ura medium. After 3 to 5 days, the PGADT7, negative and positive control vectors were introduced and cultured on SD/‐Leu. The yeast strains were picked and diluted in 0.9% NaCl to an OD_600_ of 0.1. Two μL of the suspension was spotted on a SD/‐Leu medium, with or without Aureobasidin A (ABA; Clontech). The plates were incubated for 3 to 7 days in an incubator at 30 °C.

### Transient expression in tobacco leaves

The full‐length *SPL13* ORF was amplified and cloned into the effector vector pGreen II62‐SK (Hellens *et al.*, 2005) and pHELLSGATE8. In each vector, the cauliflower mosaic virus (CaMV) 35S promoter was used to drive expression of the *SPL13* ORF. The *SFT* promoter fragment was amplified with specific primers and cloned into the reporter vectors pGreen II 0800‐LUC (Hellens *et al.*, 2005) and pMV2‐GUS, which was derived from pHellsgate8. *A. tumefaciens* GV2260 was separately transformed with the effector and reporter vectors. For transient expression experiments, young tobacco (*N. benthamiana*) leaves were co‐transformed with pSoup and the LUC vector. After two days, firefly luciferase and Renilla luciferase activity were assayed using the dual luciferase assay reagents (Promega) with an Infinite M200 (Tecan) plate reader. For GUS staining, the tobacco leaves were incubated at 37 °C for 24 h in staining buffer (100 mm sodium phosphate, pH 7, 0.1% Triton X‐100, 0.1% N‐lauroylsarcosine, 10 mm Na_2_EDTA, 1 mm K_3_Fe(CN)_6_, 1 mm K_4_Fe(CN)_6_, and 0.5 mg/mL 5‐bromo‐4‐chloro‐3‐indolyl‐β‐d‐glucuronic acid), followed by washing with 70% (v/v) ethanol. The expression of the *GUS* gene was quantified using qRT‐PCR. For the co‐immunoprecipitation assay, tobacco leaf tissue was collected and stored in liquid nitrogen. The samples were added to 1 mL of protein extraction buffer (50 mm Tris‐HCl, pH 7.5, 150 mm NaCl, 5 mm EDTA, 2 mm DTT, 10% glycerol, 1% polyvinylpolypyrrolidone, plant protease inhibitor cocktail (Sigma‐Aldrich)) to extract the total protein. The supernatant was incubated with 15 μL of anti‐FLAG affinity matrix (Roche Applied Sciences, Indianapolis, IN, USA) at 4 °C for 2 h to capture the tagged protein. Then, the matrix was washed five times with washing buffer (50 mm Tris‐HCl, pH 7.5, 250 mm NaCl, 5 mm EDTA, 10% glycerol, 1 mm PMSF), and the protein complex was analysed by SDS‐PAGE and immunoblotting. All primers used for the construction of the vectors are listed in Table S1.

### Electrophoretic mobility shift assay

The ORF of *SPL13* was amplified and inserted into pET15dMBP to generate the recombinant His‐6‐MBP‐SPL13 protein. The plasmid was introduced into *Escherichia coli* (BL21) cells as described previously (Stone *et al.*, 2005). The transformed BL21 cells were grown in 400 mL of Luria‐Bertani medium to an OD_600_ of 0.6. To induce expression, isopropyl‐β‐d‐1‐thiogalactopyranoside was added to a final concentration of 0.5 mm, and the culture was incubated at 18 °C for 3 to 5 h. Nickel‐nitrilotriacetic acid magnetic agarose (Qiagen, Germantown, MD, USA) was used to purify the recombinant protein according to the manufacturer's instructions. The proteins were analysed by Western blotting (WB) using anti‐His antibodies (MBL).

For the EMSA, we used the Light Shift Chemiluminescent EMSA Kit (Thermo Fisher Scientific, Rockford, lL, USA). Two 39‐bp single‐stranded oligonucleotide probes containing GTAC were synthesized and labelled using the Biotin 3′ End DNA Labeling Kit (Thermo Scientific). The same fragments without biotin labelling were used as competitors. The core GTAC sequence was replaced with AAGA in the mutant probe. The labelled single‐stranded oligonucleotide probes were incubated with unlabelled probes in a thermal cycler to generate labelled double‐stranded probes. The reaction conditions were 95 °C for 2 min, 75 °C for 30 s and every cycle decreasing 1 degree for 50 cycles. Then, labelled probes were incubated with the fusion protein in a 20‐μL binding reaction with or without the competitor probes at ratios of 1:1 and 1:2 for 30 min at room temperature. The protein–DNA complexes were separated with 6% native polyacrylamide gels. Finally, the biotin‐labelled probes were visualized using chemiluminescence (Chemiluminescent Nucleic Acid Detection Module; Thermo Scientific).

### Chromatin immunoprecipitation

The SPL13 protein was fused to a 6 × FLAG tag (pHELLSGATE8) and immunoprecipitated with anti‐FLAG antibodies. Chromatin immunoprecipitation was performed as described previously (Wierzbicki *et al.*, 2008; Zong *et al.*, 2016). Three grams of 2‐week‐old transgenic plant leaves were cross‐linked with 1 % (v/v) formaldehyde (0.44 m sucrose, 10 mm Tris‐HCl, pH 8.0, 10 mm MgCl_2_) for 30 min by vacuum infiltration. Glycine was added to stop the cross‐linking. The leaves were rinsed three times with ddH_2_O and ground with liquid nitrogen. The tissue powder was suspended in 25 mL of Honda Buffer (20 mm HEPES‐KOH, pH 7.4, 0.44 m sucrose, 1.25% ficoll, 2.5% Dextran T40, 10 mm MgCl_2_, 0.5% Triton X‐100, 5 mm DTT, 1 mm PMSF, 1% plant protease inhibitors (Roche)) and shaken on the rocking bed for 20 min. The lysate was filtered through two layers of Miracloth and centrifuged at 2000 × *g* for 15 min. The nuclear pellets were washed three times with 1 mL of Honda buffer and resuspended in Nuclei Lysis Buffer (50 mm Tris‐HCl, pH 8.0, 10 mm EDTA, 1% SDS, 1% Protease Inhibitors) followed by ultrasonic homogenization using a Diagenode Bioruptor (Power, high; 30s ON/30s OFF; 30 min). Next, 40 μL of protein A Dynabeads (Invitrogen) was incubated with 5 μg of anti‐FLAG antibody (ABclonal) in 1 mL ChIP dilution buffer (1.1% Triton X‐100, 1.2 mm EDTA, 16.7 mm Tris‐HCl, pH 8.0, 167 mm NaCl) at 4 ºC for 5 h on a rotating mixer. Agarose‐antibody complexes were collected using a magnet and washed three times with ChIP dilution buffer. The agarose‐antibody complexes were incubated overnight with 100 μL of chromatin supernatant from the ultrasonic homogenated in 900 μL of ChIP dilution buffer at 4 °C. A control sample (10 μL supernatant) was added to another tube as input. The antibody‐chromatin complexes were washed, eluted and de‐cross‐linked. DNA was recovered by phenol‐chloroform extraction and ethanol precipitation in the presence of glycogen and resuspended in 20 μL of distilled water. qPCR was performed to determine the relative enrichment of the different gene fragments containing GTAC sequences or on control fragments lacking the GTAC sequences. All of the primers that were used for qPCR are listed in Table S1.

### Transient Expression in tobacco Protoplasts and Microscopy

The *SPL13* CDS without the stop codon was amplified by PCR and fused to the 5′ end of the open reading frame encoding GFP in pCAMBIA 1302, which uses the CaMV 35S promoter to drive expression. Hereafter, we refer to this plasmid as 35S:SPL13‐GFP. Ghd7‐CFP was used as the marker for the nucleus. Selected tobacco (*N. benthamiana*) seedlings leaves were cut into 1–1.5 mm strips and incubated with the enzyme digestion solution (1.5% Cellulase R10, 0.4% Macerozyme R10, 0.1% BSA, 20 mm MES, 10 mm CaCl_2_, 20 mm KCl, 0.4 m Mannitol) for 1 h using vacuum infiltration and then incubated in the dark for 4 h. An equal volume of W5 solution (2 mm MES, 154 mm NaCl, 125 mm CaCl_2_, 5 mm KCl) was added. Digested tissues were filtered through Miracloth. After centrifugation at 150 × *g* for 2 min at 4 °C, the supernatant was diluted with 5 mL of W5 solution. Ten microgram of the SPL13‐GFP and Ghd7‐CFP plasmids was added to 100 μL of protoplasts and incubated with 110 μL of PEG transfection solution (0.2 m Mannitol, 100 mm CaCl_2_, 40% PEG 4000) for 10 min at room temperature. A total of 420 μL of W5 solution was added to stop the transfection. The protoplasts were centrifuged at 100 × *g* for 2 min at room temperature using a bench‐top centrifuge, and the supernatant was removed. The protoplasts were gently resuspended with 500 μL of WI solution (4 mm MES, 20 mm KCl, 0.5 m Mannitol) and sonicated as described previously (Yoo *et al.*, 2007). After incubating the samples for 12–16 h at 28 °C, fluorescence from the transformed protoplasts was imaged using a confocal laser scanning microscope (Leica TCS SP2). The pertinent primer sequences are listed in Table S1.

### 5′ RACE analysis of 35S‐miR156a plants

Total RNA was extracted from 35S‐miR156a plants using the TRIzol reagent (Invitrogen). Cleaved mRNAs were self‐ligated to their poly(A) tails to yield circular RNAs using T4 RNA Ligase 1 (20 units ssRNA Ligase, 1× T4 RNA Ligase buffer, 1 mm ATP, 3 μg RNA). Complementary DNAs were synthesized using the M‐MLV reverse transcriptase kit (Toyobo). The cleaved fragments of the *SPL13* gene were amplified using nested PCR. The PCR products were ligated into the *pEASY*‐Blunt Cloning Vector (TransGene Biotech, Haidian District, Beijing, China) and analysed using the M13 primer.

### Statistical analysis

Statistical analyses utilized SigmaPlot, Excel and the SPSS software, New York, NY, USA. Comparisons between pairs of the groups were performed using Student′s *t*‐test. Statistically significant differences were categorized into two groups: *P* < 0.05 and *P* < 0.01.

### Accession Numbers

The gene sequences used in our experiments are available from the GenBank databases using the following accession numbers: *SlSPL13*, Solyc05g015840; *SlSPL2*, Solyc05g015510; *SlCNR*, Solyc02g077920; *SlSPL3*, Solyc10g009080; *SlSPL6a*, Solyc03g114850; *SlSPL6b*, Solyc05g012040; *SlSPL15*, Solyc10g078700; *SlSFT*, Solyc03g063100; *SlAP1*, Solyc05g056620; *SlSP*, Solyc06g074350; *SlFA*, Solyc03g118160.2; *SlTMF*, Solyc09g090180.1; *SlAN*, Solyc02g081670.1 and *SlJOINTLESS*, Solyc11g010570.2.

## Conflicts of interest

The authors declare no competing financial interests.

## Author contributions

J.Z. and Z.Y. planned and designed the research. L.C., F.Z., J.W., C.Z., J.Y. and C.L. performed the experiments, conducted fieldwork, analysed data and made conclusions based on the results. L.C., F. X. and J.Z. wrote the manuscript.

## Supporting information


**Figure S1 **Phenotypes of the SPL13‐overexpressing (pHELLSGATE8, 35S‐SPL13, T0 generation) and SPL13‐RNAi transgenic tomato plants.
**Figure S2 **Phenotype of CR‐spl13 lines.
**Figure S3 **Targeting of SPL13 by miR156a *in vivo*.
**Figure S4 **Purification and analysis of the recombinant SPL13 protein.
**Figure S5 **Phenotypes of transgenic plants harboring the 35S‐SPL13‐FLAG (pHELLSGATE8) transgene. Red arrows indicate the leaf axils. The accumulation of the SPL13‐FLAG fusion protein in the transgenic plants but not in WT was verified by western blotting using anti‐FLAG antibodies. The large subunit of rubisco was used as a loading control.
**Figure S6 **Phylogeny of the SPL gene family in Arabidopsis, rice and tomato.
**Figure S7 **Amino acid sequence alignment of proteins encoded by seven miR156‐targeted SPL genes.
**Figure S8 **Expression patterns of six miR156a‐targeted SPL genes in CR‐spl13 and WT tomato plants.
**Figure S9 **Expression of SlSFT, SlFA and SlAP1 in spl13 mutant lines and WT.
**Figure S10 **Expression of SP, JOINTLESS, TMF and AN in spl13 mutant lines and WT.
**Figure S11 **Inability of SPL13 to bind the SP promoter.
**Table S1 **Sequences of primers used in this study.
