# Supplementary material for: miR156a‐targeted SBP‐Box transcription factor SlSPL13 regulates inflorescence morphogenesis by directly activating SFT in tomato
Source: Plant Biotechnol J. 2020 Jan 25;18(8):1670–82. doi: 10.1111/pbi.13331 (PMC7336387; doi:10.1111/pbi.13331)
Supplement: Supplementary file 1 — Figure S1 Phenotypes of the SPL13‐overexpressing (pHELLSGATE8, 35S‐SPL13, T0 generation) and SPL13‐RNAi transgenic tomato plants. Figure S2 Phenotype of CR‐spl13 lines. Figure S3 Targeting of SPL13 by miR156a in vivo. Figure S4 Purification and analysis of the recombinant SPL13 protein. Figure S5 Phenotypes of transgenic plants harboring the 35S‐SPL13‐FLAG (pHELLSGATE8) transgene. Red arrows indicate the leaf axils. The accumulation of the SPL13‐FLAG fusion protein in the transgenic plants but not in WT was verified by western blotting using anti‐FLAG antibodies. The large subunit of rubisco was used as a loading control. Figure S6 Phylogeny of the SPL gene family in Arabidopsis, rice and tomato. Figure S7 Amino acid sequence alignment of proteins encoded by seven miR156‐targeted SPL genes. Figure S8 Expression patterns of six miR156a‐targeted SPL genes in CR‐spl13 and WT tomato plants. Figure S9 Expression of SlSFT, SlFA and SlAP1 in spl13 mutant lines and WT. Figure S10 Expression of SP, JOINTLESS, TMF and AN in spl13 mutant lines and WT. Figure S11 Inability of SPL13 to bind the SP promoter. Table S1 Sequences of primers used in this study. [file PBI-18-1670-s001.pdf]

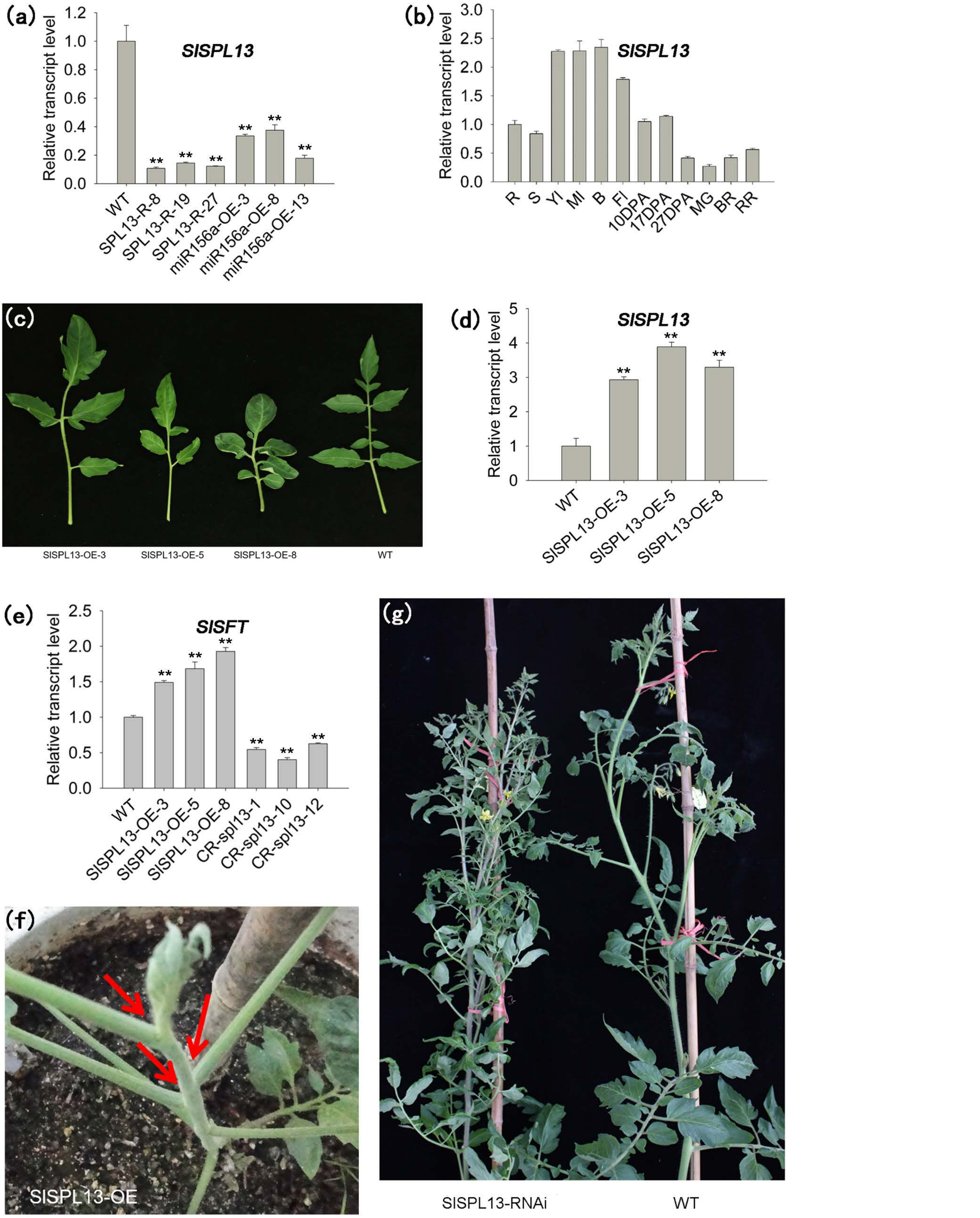

Figure S1 Phenotypes of the *SPL13*-overexpressing (pHELLSGATE8, 35S-SPL13, T0 generation) and *SPL13*-RNAi transgenic tomato plants. (a) Quantitative RT-PCR analysis of *SPL13* expression in the young leaves of transgenic and WT tomato plants. The tissues were collected immediately after planting. Three replicate experiments were performed. The bars represent mean values ± SE. Asterisks indicate statistically significant differences relative to the wild type and were determined using *t*-tests. **, $P < 0.01$. (b) The transcript levels of *SPL13* in different tomato organs: R, root; S, stem; Yl, young leaf; Ml, mature leaf; B, bud; Fl, flower; 10 DPA, ten days post anthesis; 17 DPA, seventeen days post anthesis; 27 DPA, twenty-seven days post anthesis; MG, mature green stage fruit; BR, breaker stage fruit; RR, red ripe stage fruit. All samples were collected at nine weeks after planting. (c) Leaf phenotypes of the *SPL13*-overexpressing transgenic tomato lines. (d, e) Quantitative RT-PCR analysis of *SPL13* and *SFT* expression in the young leaves of three transgenic lines and WT. Three replicate experiments were performed. The bars represent mean values ± SE. Asterisks indicate statistically significant differences relative to the wild type and were determined using *t*-tests. **, $P < 0.01$. (f) Lateral branching phenotype of the *SPL13*-overexpressing line grown in the greenhouse. Red arrows represent the leaf axils. (g) Lateral branching phenotypes of the *SPL13*-RNAi line and WT tomato grown in the greenhouse.

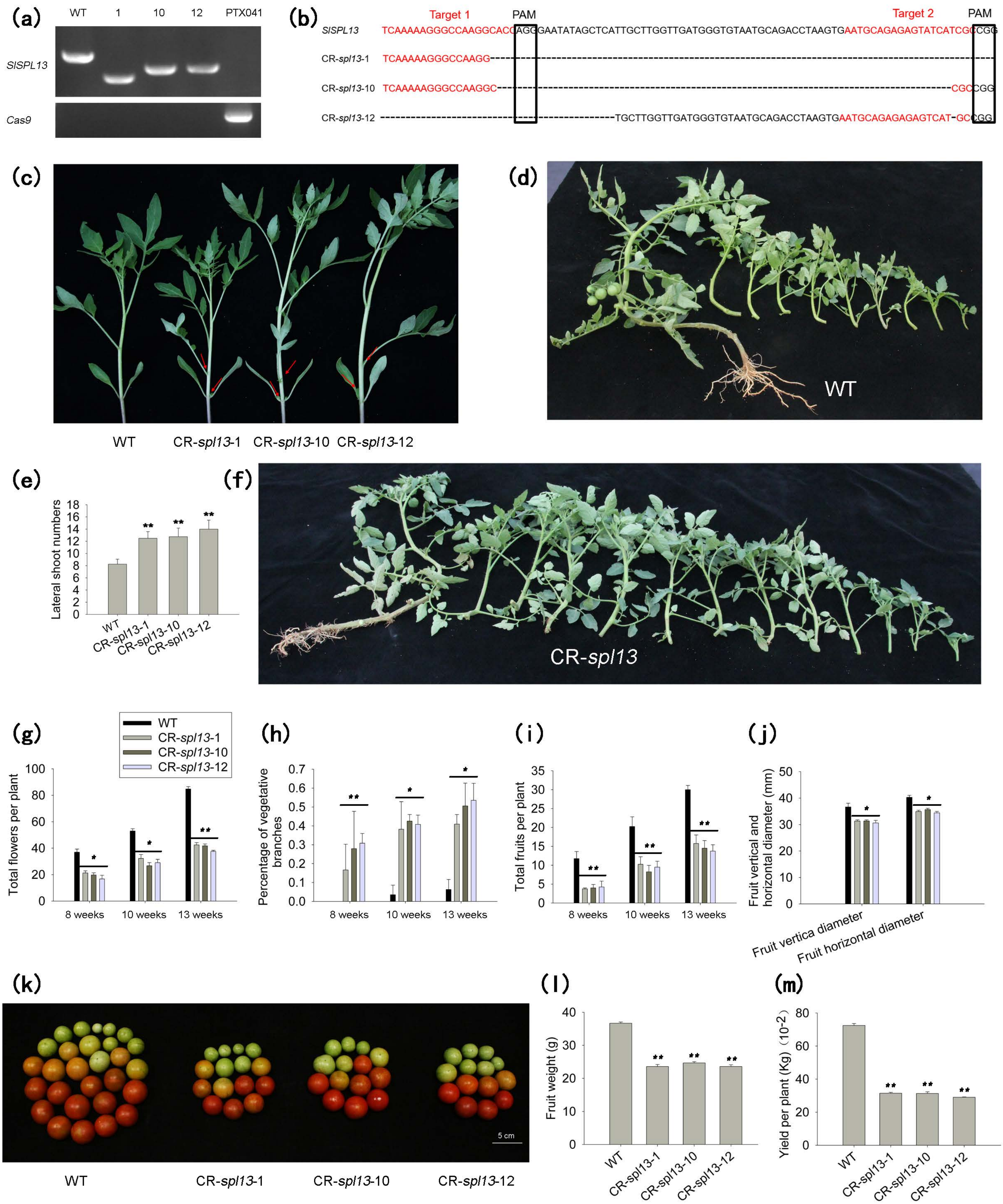

Figure S2 Phenotype of CR-*spl13* lines. (a) PCR-based analysis of three CR-*spl13* mutant alleles with different amplicon lengths. The empty PTX041 plasmid served as a positive control and is also shown. (b) Verification of the CR-*spl13* mutant alleles by DNA sequencing analysis. The red font indicates sgRNA target sequences. The black boxes indicate the protospacer-adjacent motif (PAM) sequences. (c) Lateral branching of the leaf axils in the CR-*spl13* tomato plants. Lateral branches are indicated with red arrows. (d) Lateral branching in WT tomato plants. (e) Lateral branching numbers of the CR-*spl13* and WT tomato plants. (f) Lateral branching in CR-*spl13* tomato plants. (g-i) Accumulation of flowers, fruit and the percentage of vegetative branch inflorescences per plant at three developmental stages. (j) Statistical comparison of the vertical and horizontal diameters of the fruit from CR-*spl13* and WT tomato plants. (k) Total fruit yield per plant for WT (right) and representative CR-*spl13* (left) tomato plants. (l, m) Mean values for the total fruit yield and fruit weight from the CR-*spl13* and WT tomato plants. Three transgenic lines from four representative transgenic plants and four representative WT plants were selected for statistical comparisons. Asterisks indicate statistically significant differences relative to the WT and were determined using t-tests. *, $P < 0.05$, **, $P < 0.01$.

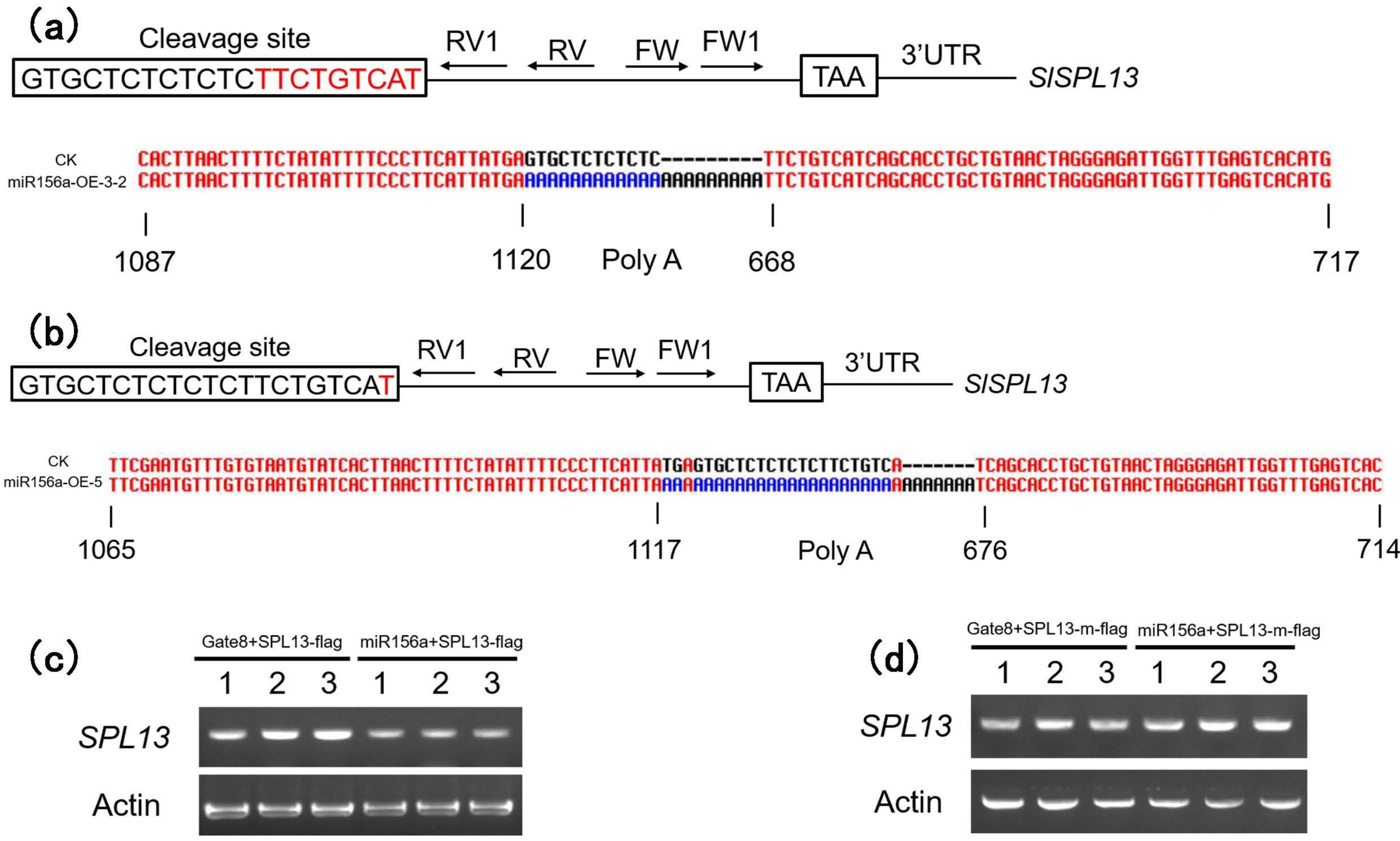

Figure S3 Targeting of SPL13 by miR156a *in vivo*. (a, b) Cleavage site analysis of SPL13 mRNA in miR156a-OE-3-2 and miR156a-OE-5. The products were ligated into the vector. The sequences detected using the M13 primer are shown. (c, d) Transient co-expression of FLAG-tagged SPL13, SPL13-mutant (SPL13-m) and 35S-miR156a in the leaves of *N. benthamiana*. FLAG-tagged SPL13, SPL13-mutant (SPL13-m) and the empty vector (pHELLSGATE8) were transiently co-expressed. Leaf extracts were analyzed by using RT-PCR. These experiments were repeated three times and yielded similar results each time.

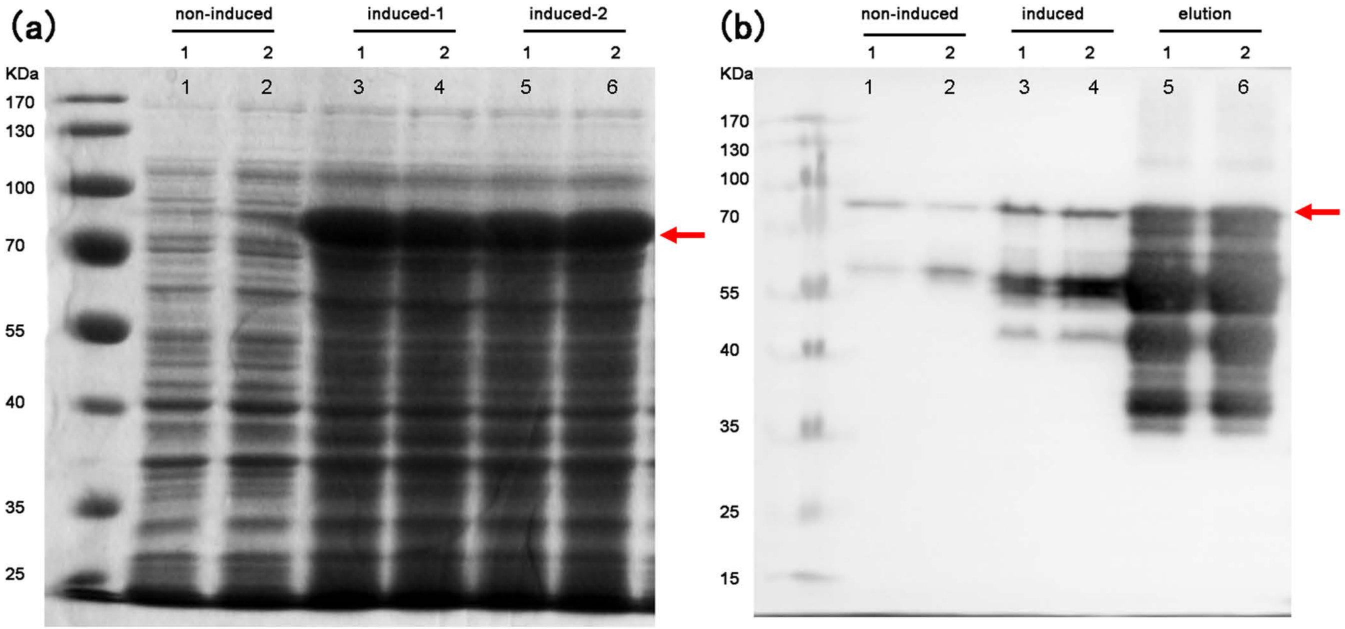

Figure S4 Purification and analysis of the recombinant SPL13 protein. (a) Analysis of recombinant SPL13 proteins using SDS-PAGE. The SDS gels were stained with Coomassie blue. Lanes 1 and 2, non-induced; 3-6, induced. The red arrow indicates the His-6-MBP-SPL13 fusion protein. (b) Analysis of recombinant SPL13 proteins using western blotting. The proteins were analyzed using SDS-PAGE and western blotting with anti-His antibodies. Lanes 1 and 2, not induced; 3 and 4, induced; 5 and 6, elution. The red arrow indicates the His-6-MBP-SPL13 fusion protein.

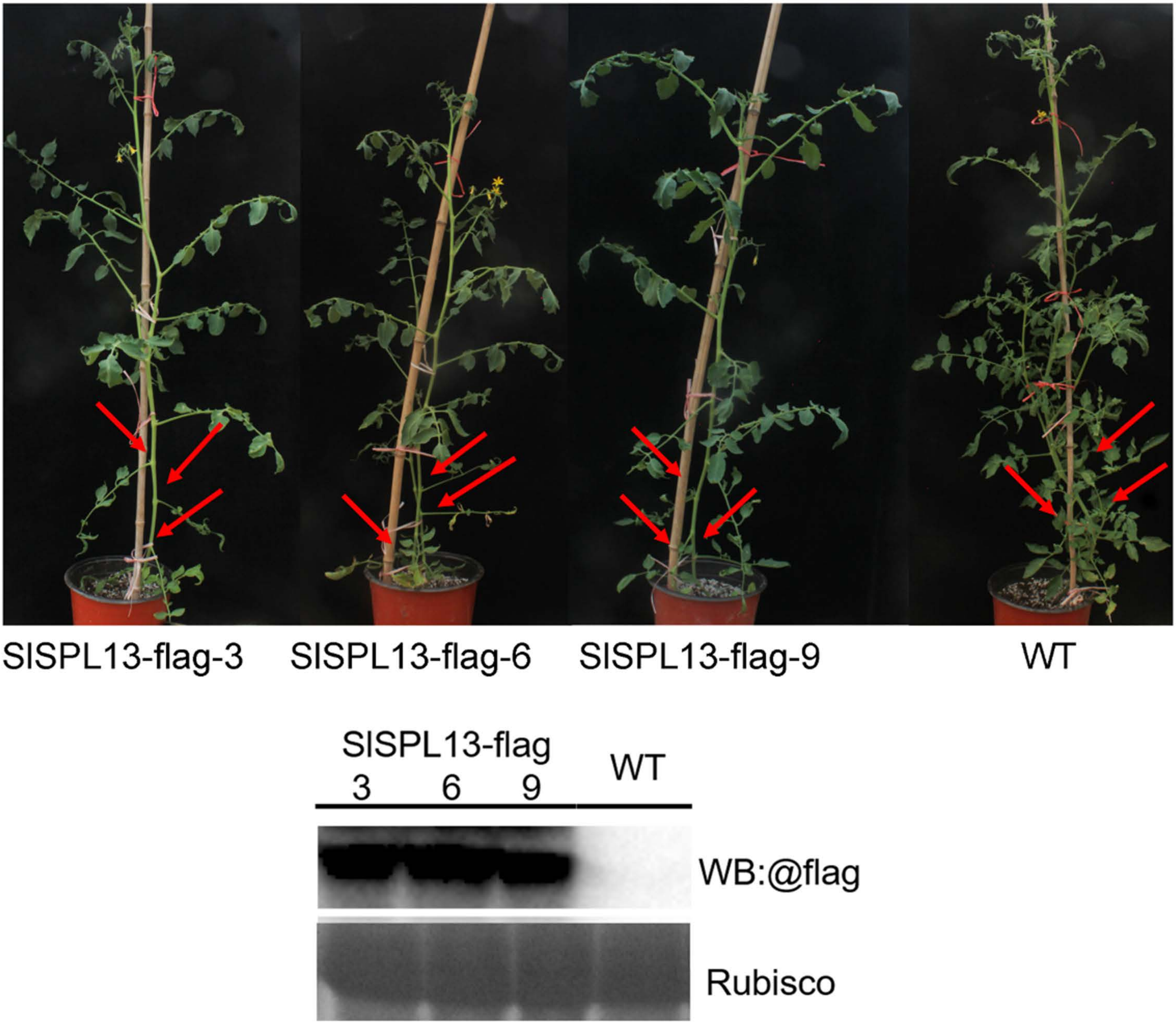

Figure S5 Phenotypes of transgenic plants harboring the 35S-SPL13-FLAG (pHELLSGATE8) transgene. Red arrows indicate the leaf axils. The accumulation of the SPL13-FLAG fusion protein in the transgenic plants but not in WT was verified by western blotting using anti-FLAG antibodies. The large subunit of rubisco was used as a loading control.

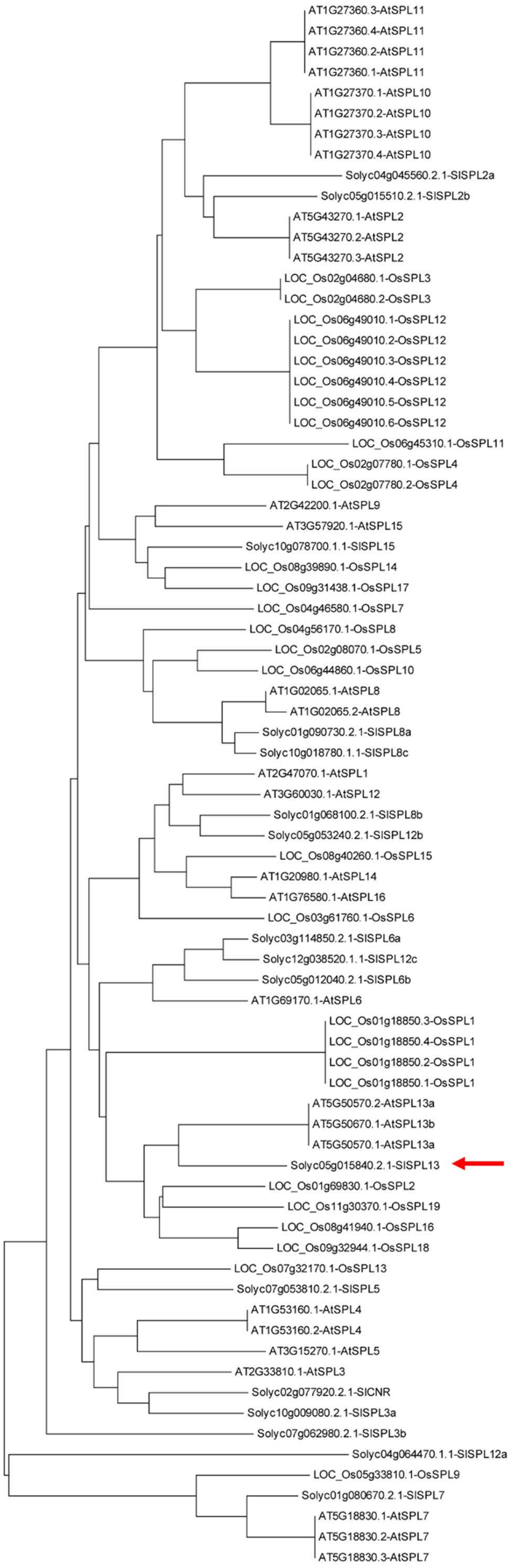

Figure S6 Phylogeny of the *SPL* gene family in *Arabidopsis*, rice and tomato.

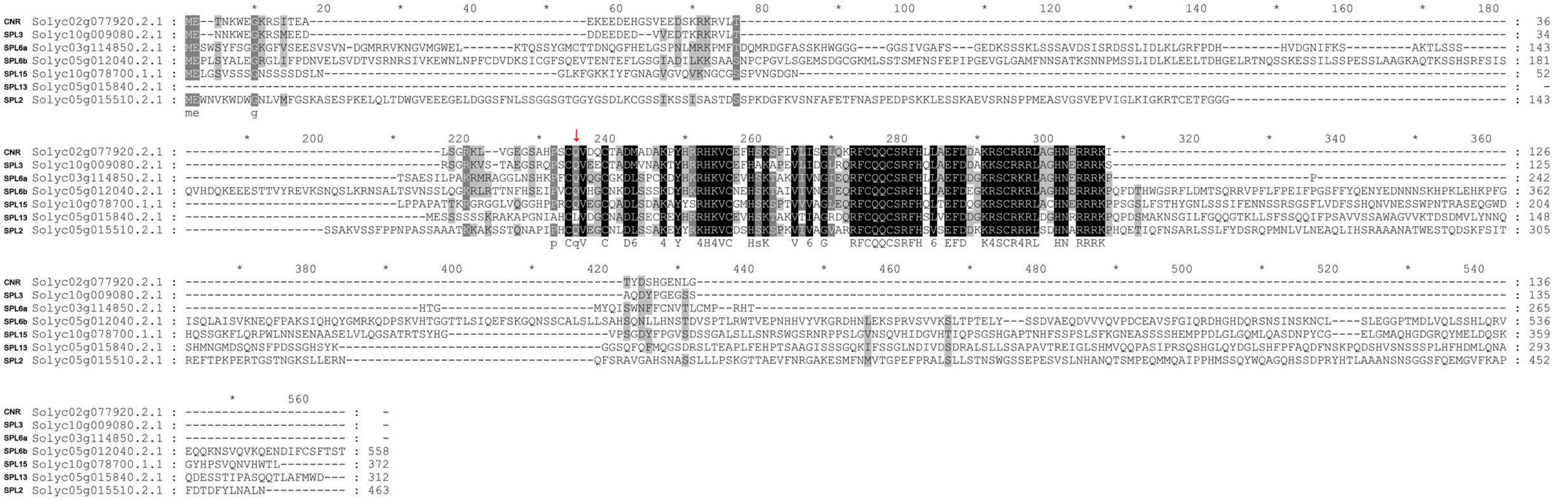

Figure S7 Amino acid sequence alignment of proteins encoded by seven miR156-targeted *SPL* genes.

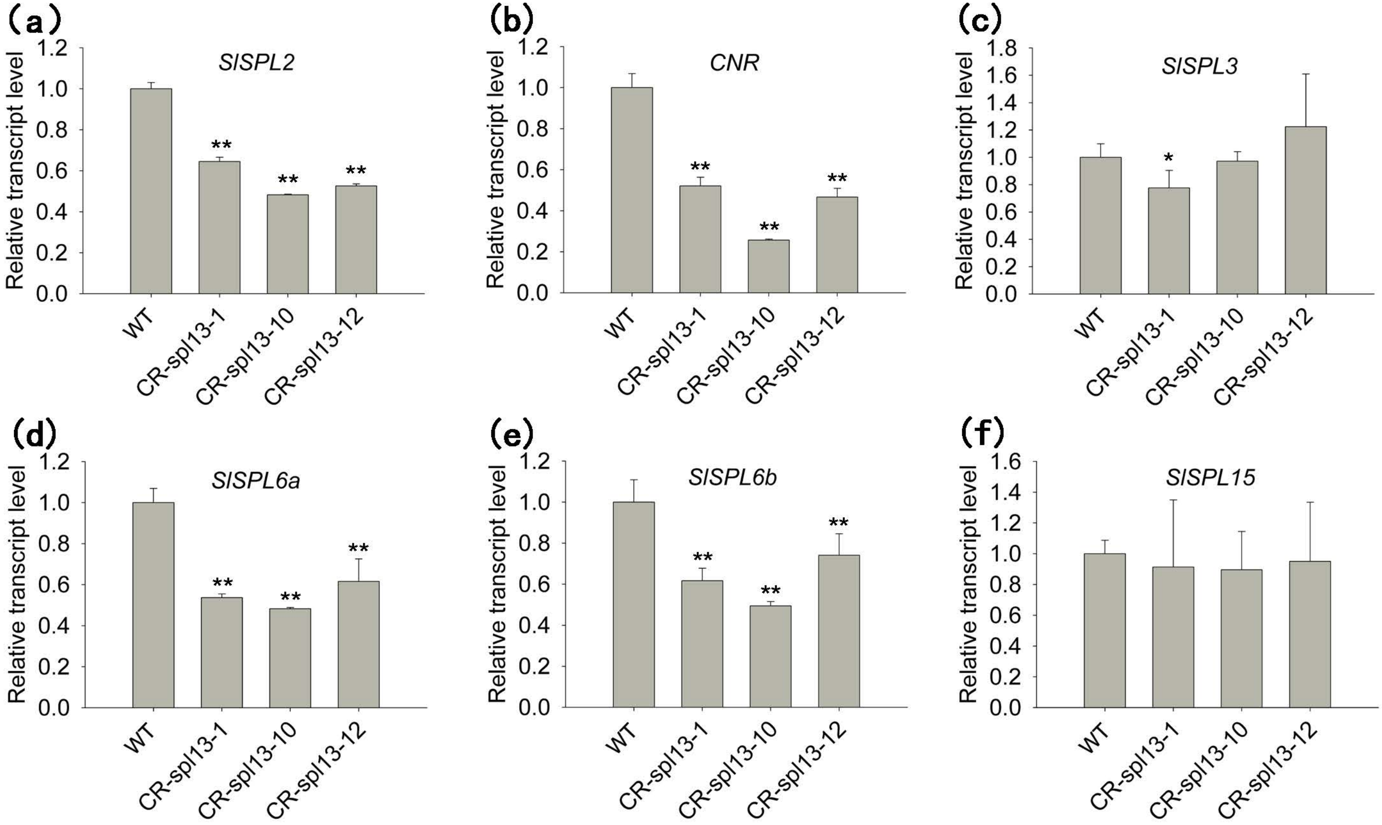

Figure S8 Expression patterns of six miR156a-targeted *SPL* genes in CR-*spl13* and WT tomato plants. (a-f) Quantitative RT-PCR analysis of *SlSPL2*, *CNR*, *SlSPL3*, *SlSPL6a*, *SlSPL6b* and *SlSPL15* expression in young leaves of three CR-*spl13* lines and WT tomato plants. Three replicate experiments were performed. The data presented are the means ± SE. Asterisks indicate statistically significant differences relative to the wild type and were determined using *t*-tests. *, $P < 0.05$, **, $P < 0.01$, no asterisks, $P > 0.05$.

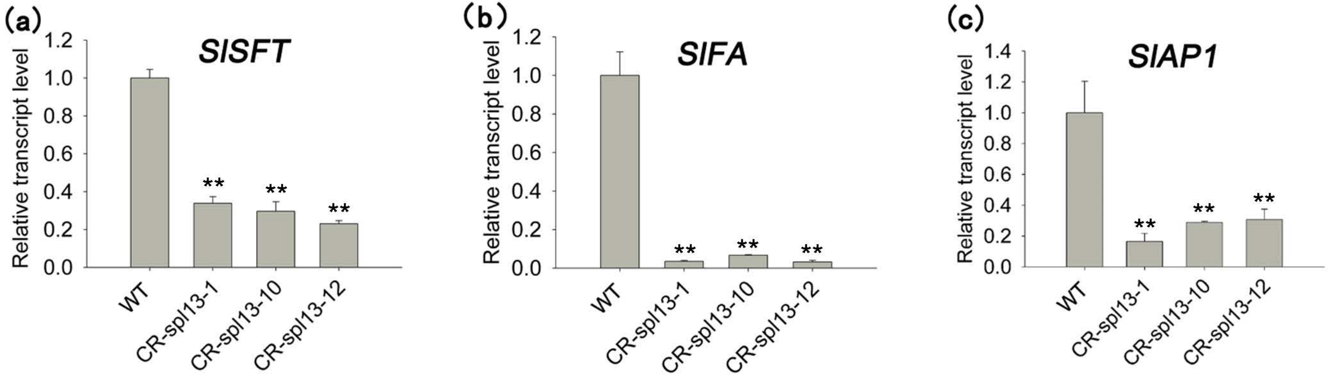

Figure S9 Expression of *SlSFT*, *SlFA* and *SlAP1* in *spl13* mutant lines and WT. (a-c) Quantitative RT-PCR analysis of *SlSFT*, *SlFA* and *SlAP1* expression in the young tissues of *spl13* mutant lines lacking the PTX vector and in WT tomato plants. Three replicate experiments were performed. The data presented are the means ± SE. Asterisks indicate statistically significant differences relative to the wild type and were determined using *t*-tests. **, $P < 0.01$.

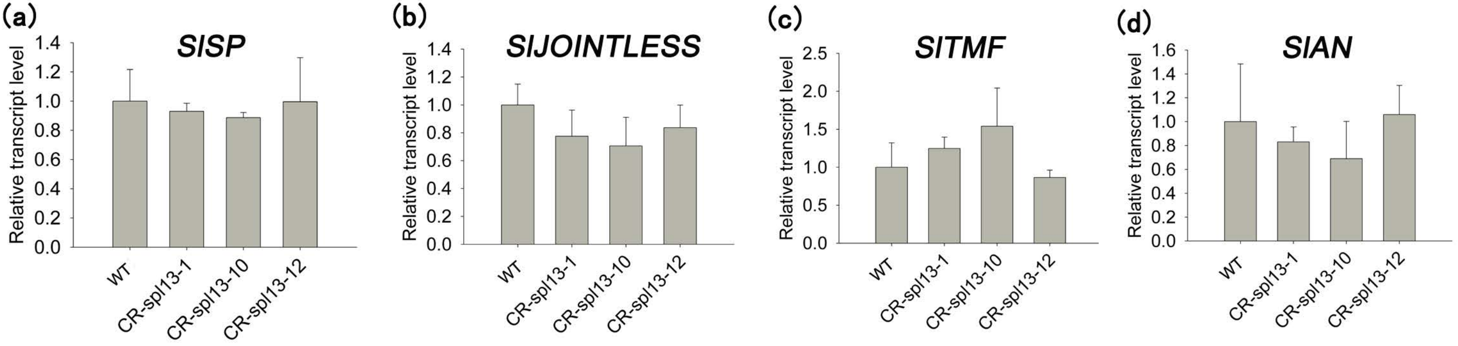

Figure S10 Expression of *SP*, *JOINTLESS*, *TMF* and *AN* in *spl13* mutant lines and WT. (a-d) Quantitative PCR analysis of *SP*, *JOINTLESS*, *TMF* and *AN* expression in the young tissues of *spl13* mutant lines without the PTX vector and in WT tomato plants. Three replicate experiments were performed. The data presented are the means ± SE. Asterisks indicate statistically significant differences relative to the wild type and were determined using *t*-tests. *, $P < 0.05$, **, $P < 0.01$, no asterisks, $P > 0.05$.

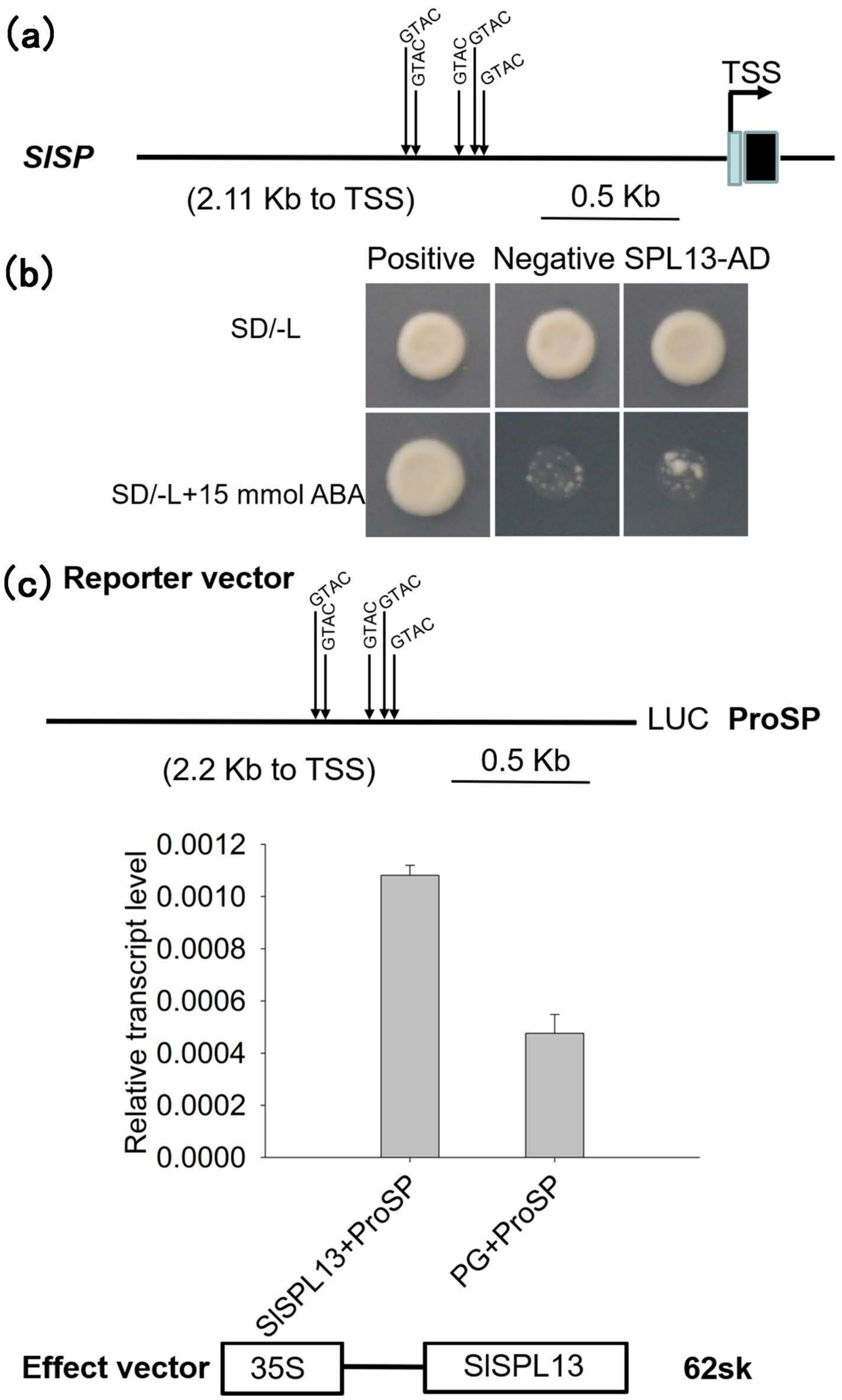

Figure S11 Inability of SPL13 to bind the *SP* promoter. (a) Schematic diagram of the *SP* promoter region (2185 bp). Four *cis*-elements were identified in the promoter of *SP*. The promoter fragment (-1213 to -846 from the start codon) was used in the Y1H assay. (b) Failure of SPL13 to bind the *SP* promoter fragment in the Y1H assay. The bait vector containing SP and the prey vector containing SPL13 were introduced into the yeast strain Y1H gold. Enhanced resistance to ABA indicates interactions between the bait and prey. Co-transformations of the bait vector containing SP with pGADT7 or pGADT-Rec2-53 were used as negative and positive controls, respectively. (c) Dual luciferase system-based analysis of SPL13 binding *SP* promoters. The *SP* promoter fragment was inserted into the reporter vector (pGreen II 0800 LUC) and SPL13 was inserted into the effector vector (pGreen II 62-SK). We used Agrobacterium tumefaciens-mediated transformation to transiently express these constructs in tobacco (*Nicotiana benthamiana*) leaves. LUC, firefly luciferase activity; RLU, Renilla luciferase activity; PG, the empty vector of pGreen II 62-SK. PG served as a control. Values are presented as means ± SE (n = 3).

**Table S1. Primers used in this study.**	
**Primer name**	**Sequence (5'-3')**
SlSBP13-DT1-FW	GAATCTAACAGTGTAGTTTGTCAAAAAGGGCCAAGGCACCGTTTTAGAGCTAGAAATAG
SlSBP13-DT2-RV	GCTATTTCTAGCTCTAAAACGCGATGATACTCTCTGCATTCAAACTACACTGTTAGATT
SlSBP13-RNAi-FW	AAAAAGCAGGCTAATCATCATCATCGTCATCAAAAAG
SlSBP13-RNAi-RV	AGAAAGCTGGGTCTGCTAAATGACAGGAGTTTCGTT
SlSBP13-OE-FW	AAAAAGCAGGCTTAGAGGCAAAAATTGAATTGTGTG
SlSBP13-OE-RV	AGAAAGCTGGGTCATTTGGTTGGATGGATTGAAG
SlSPL2-OE-FW	AAAAAGCAGGCTCCCAAGATGGAGTGGAATGTG
SlSPL2-OE-RV	AGAAAGCTGGGTCATCATACTAACAGACCTGCTTCATC
SlSPL2-RNAi-FW	AAAAAGCAGGCTTCTACTGACTCTTCACCAAAGGATG
SlSPL2-RNAi-RV	AGAAAGCTGGGTTGACAGTGAGGAATTGGTGCAT
SlCNR-OE-FW	AAAAAGCAGGCTGTCTGGCTTCCTCACTCTATTTT
SlCNR-OE-RV	AGAAAGCTGGGTCATGCTTAACCCACATCTTGT
SlCNR-RNAi-FW	AAAAAGCAGGCTGCAAACAGAGATTATCCTTCAGA
SlCNR-RNAi-RV	AGAAAGCTGGGTCATGCTTAACCCACATCTTGT
SlSPL3-OE-FW	AAAAAGCAGGCTGTTCCGAGTAACTCAATCTATCCATC
SlSPL3-OE-RV	AGAAAGCTGGGTTATGCCATCTGCCGTGCTACT
SlSPL3-RNAi-FW	AAAAAGCAGGCTACTTATTGATGGACTCCGACAGC
SlSPL3-RNAi-RV	AGAAAGCTGGGTGAGGGAGCTTAAGTTCTCATCAGTC
SlSPL6a-OE-FW	AAAAAGCAGGCTTTTCTCCTTTCCCAAACCACC
SlSPL6a-OE-RV	AGAAAGCTGGGTTACTCCCTGATACTTCCACCTCTGT
SlSPL6a-RNAi-FW	AAAAAGCAGGCTCTCCTCTTCTTCAGTGCTCCTTG
SlSPL6a-RNAi-RV	AGAAAGCTGGGTATTCCTCTGATACAAAACCCTTCC
SlSPL6b-OE-FW	AAAAAGCAGGCTAAAGCACCATAGCACCTGTTC
SlSPL6b-OE-RV	AGAAAGCTGGGTATGATGCAAGATTCCTTATAGACAA
SlSPL6b-RNAi-FW	AAAAAGCAGGCTAAAGCACCATAGCACCTGTTC
SlSPL6b-RNAi-RV	AGAAAGCTGGGTGAGAATGCGAACTCTTTGTTTG
SlSPL15-OE-FW	AAAAAGCAGGCTTTTTGAACCTATAATGGAACTGG
SlSPL15-OE-RV	AGAAAGCTGGGTTCATAAACGAAGGGGAGGAC
SlSPL15-RNAi-FW	AAAAAGCAGGCTGGTTCAGTGTCTTCTTCGGGTAA
SlSPL15-RNAi-RV	AGAAAGCTGGGTCTACACTGTTGGCAAAACCTCTG
SPL13-LIC6FLAG-FW	GACGATGACAAGGAATTCATGGAATCATCATCATCGTCATC
SPL13-PH7LIC-RV	ATAGGGAAGAGGCCTGAATTCGTCCCACATAAAGGCTAGTGTTTG
SPL13-62SK-FW	GCCGCTCTAGAACTAGTGGATCCTAGAGGCAAAAATTGAATTGTGTG
SPL13-62SK-RV	TTGGTACCGGGCCCCCCCTCGAGCATTTGGTTGGATGGATTGAAG
SPL13-AD-FW	ACGTACCAGATTACGCTCATATGTAGAGGCAAAAATTGAATTGTGTG

SPL13-AD-RV	TACGATTCATCTGCAGCTCGAGCCATTTGGTTGGATGGATTGAAG
SPL13-mutant-FW	ACGGGGGACTCTTGACCATGGTAATGGAATCATCATCATCGTCATC
SPL13-mutant-RV	AAGTTCTTCTCCTTTACTAGTGTCCCACATAAAGGCTAGTGTTTG
SPL13-mutant-RVF	GATAGAAGCGACAGGGCACGATCAGAATCAACAATATCATTTAATCC
SPL13-mutant-FWR	GGATTAAATGATATTGTTGATTCTGATCGTGCCCTGTCGCTTCTATC
SPL13-PET15d-MBP-FW	TCTGTTCCAGGGGCCGCATATGATGGAATCATCATCATCGTCATC
SPL13-PET15d-MBP-RV	TGTTAGCAGCCGGATCCTCGAGTTAGTCCCACATAAAGGCTAGTGTT
SPL13-6FLAG-FW	GATGACGATGACAAGGAATTCATGGAATCATCATCATCGTCATC
SPL13-6FLAG-RV	GTCCTTGTAATCCATGAATTCGTCCCACATAAAGGCTAGTGTTTG
P1-FW	TTAAGTAAATAATTGTGACCCCGAG
P1-RV	CGATCTAGTCCCAAGAATGAATGT
P2-FW	CATTCATTCTTGGGACTAGATCGT
P2-RV	TCGCATGTGCTCAATAAGAAAAG
P3-FW	CTATGGTTGTAAGGTGGGAAAAGAG
P3-RV	ATACACATATGGTAGCACCTTTTGC
P4-FW	CTTGTAAAAATATGCATAGCCCG
P4-RV	GGCTAACTTTTGTGTATTTGTGAGC
CK1-FW	TACTTTACGTGATGTATCAGTCGGA
CK1-RV	CATCGCATACGTCCAGATAGAAC
CK2-FW	GAAATAGTGAGCTATGAAAGTCCAA
CK2-RV	CATACACTGTTTGCCGACCT
SFT-Q-FW	CGAGCTTAGGCCTTCCCAA
SFT-Q-RV	CATACACTGTTTGCCGACCT
AP1-Q-FW	TTCGATCGAGAAAGAACCAA
AP1-Q-RV	TTAGTTTGCTGGTGCCATTC
SPL13-Q-FW	TTCACATGTCAGCAACAGCA
SPL13-Q-RV	CCCACATAAAGGCTAGTGTTTGCTG
SPL2-Q-FW	CCCCTTGTCCACTCTAAACCTAC
SPL2-Q-RV	GTCTGCATCTCAGTGGTCCCT
CNR-Q-FW	GCTAAGAGGAGTTGCCGAAGG
CNR-Q-RV	GGCTGCCATTGATGCTGAT
SPL3-Q-FW	ATGGACTCCGACAGCGTTTC
SPL3-Q-RV	CCAACCGTCTCCTGCAACTC
SPL6a-Q-FW	CTCAACTTGCCATGCCCTTTA
SPL6a-Q-RV	CTTGTTGTTACAGCAGGTAGTCCA
SPL6b-Q-FW	TCAACATCCGCAATGAAGAATC
SPL6b-Q-RV	AAAGCTATGGCTGACACTTACAAA

SPL15-Q-FW	CCTGGAGTCTCAGATTCAAGTGG
SPL15-Q-RV	GAAGGGCTTGAGAAGTGATTGG
SlSBP13-DET-FW	CTTGGAGTAGGTGGTGGATGG
SlSBP13-DET-RV	AAGGTACTGGGTTCATCCAAATC
Sly-mir156a-FW	CATTTGGAGAGGACACGCTCGAGAAAATCTCTAATTTAGTTGTTTGTTTTTTG
Sly-mir156a-RV	TCTCATTAAAGCAGGACCTCGAGAGATTTAAATATTTTTACGAAAGAGGTG
SPL13-race-FW1	GAATCATCTACAATCCCTGCCTC
SPL13-race-FW	GATTCACATGTCAGCAACAGCAG
SPL13-race-RV	CTTAGACCATCATACTGCAGGCC
SPL13-race-RV1	CATGTGACTCAAACCAATCTCCC
M13-FW	GTAAAACGACGGCCAG
M13-RV	CAGGAAACAGCTATGAC
SFT-0800-FW	CACTATAGGGCGAATTGGGTACCGTGATTGTTCATCTATGTGTTCCTGT
SFT-0800-RV	TATGTTTTTGGCGTCTTCCATGGGACGATGGTTGACGATAAACAAA
PSFT-GUS-FW	TGCATCCAACGCGTTGGGAGCTCGTGATTGTTCATCTATGTGTTCCTGT
PSFT-GUS-RV	GCCTTCGCCATTCTAGACTCGAGGACGATGGTTGACGATAAACAAA
GUS-Q-FW	AAGTGTCGGTCGTGGATGAG
GUS-Q-RV	AGTCCGTCGTTCACCAGTTC
SFT1- PAbai-FW	AGCTTGAATTCGAGCTCGGTACCGTCATGATTCGATTAGAAGAATTCCT
SFT1- PAbai-RV	ACATACAGAGCACATGCCTCGAGGACGATGGTTGACGATAAACAAA
SFT2- PAbai-FW	AGCTTGAATTCGAGCTCGGTACCGTGATTGTTCATCTATGTGTTCCTGT
SFT2- PAbai-RV	ACATACAGAGCACATGCCTCGAGGGAATGAAACCCCAATCAAGAA
Sft-Probe1-FW	TAACTAGCTAGCTAGGAGTACTCTTGTGTTGTGTTTTAG
Sft-probe1-RV	CTAAAACACAACACAAGAGTACTCCTAGCTAGCTAGTTA
Sft-mProbe1-FW	TAACTAGCTAGCTAGGAAAGATCTTGTGTTGTGTTTTAG
Sft-mProbe1-RV	CTAAAACACAACACAAGATCTTTCCTAGCTAGCTAGTTA
Sft-Probe2-FW	ATTTTGTAATAAAATATTGTACTTGCTCTTTTCTTATTG
Sft-probe2-RV	CAATAAGAAAAGAGCAAGTACAATATTTTATTACAAAAT
Sft-mProbe2-FW	ATTTTGTAATAAAATATTAAGATTGCTCTTTTCTTATTG
Sft-mProbe2-RV	CAATAAGAAAAGAGCAATCTTAATATTTTATTACAAAAT
SP- PAbai-FW	AGCTTGAATTCGAGCTCGGTACCGTCACATAAAATGAGACGGAAAAAG
SP- PAbai-RV	ACATACAGAGCACATGCCTCGAGGAACGTTACGCTAATGGTGAAGATA
SP-0800-FW	CACTATAGGGCGAATTGGGTACCCTGCCAGCTTGAGGGGGA
SP-0800-RV	TATGTTTTTGGCGTCTTCCATGGTTTGGAAGCCATATATATATTTAGAGTTAA
SP-Q-FW	GGGTTGAAGTTCATGGTGGT
SP-Q-RV	CCCAACCACTTCTCTTCCAA
FA-Q-FW	GCTCCCAACATCATCCTACTCC

FA-Q-RV	CGCTTTGATACCGTACCTCTCTC
AN-Q-FW	GCTTTCACTTTCCCTATGCTTTTC
AN-Q-RV	CTCTAGCTCTAAAGAAAGCAGGTGG
J-Q-FW	CTTTCTGTTCTCTGTGATGCTGATG
J-Q-RV	GTCCTTGAAGTTCTTCTCCCCTC
TMF-Q-FW	CCTAAGAAACCATAGACCACCACTC
TMF-Q-RV	GGGTTTTCCACCGTTTTCC
SPL13-mutant-Q-FW	TTCACATGTCAGCAACAGCAGCA
SPL13-mutant-Q-RV	CACATAAAGGCTAGTGTTTGCTGAG
SPL13-LIC6FLAG-m-FW	GACGATGACAAGGAATTCATGGAATCATCATCATCGTCATC
SPL13-PH7LIC-m-RV	ATAGGGAAGAGGCCTGAATTCGTCCCACATAAAGGCTAGTGTTTG
SPL13-RTPCR-FW	CAAAGTCACAATTGCAGGTCG
SPL13-RTPCR-RV	GGAACGAGTTTTGACTGTCCATAC
β-actin-Fw	ATGGCAGACGGAGAGGATATTCA
β-actin-Rv	GCCTTTGCAATCCACATCTGCTG
